# The gut microbiota-inflammation-HFpEF axis: deciphering the role of gut microbiota dysregulation in the pathogenesis and management of HFpEF

**DOI:** 10.3389/fcimb.2025.1537576

**Published:** 2025-03-13

**Authors:** Shenghua Zhou, Xuan Zhou, Panpan Zhang, Wei Zhang, Jinli Huang, Xuzhao Jia, Xiaole He, Xin Sun, Hui Su

**Affiliations:** ^1^ Department of Geriatrics, Xijing Hospital, the Fourth Military Medical University, Xi’an, China; ^2^ Department of Pediatrics, Xijing Hospital, the Fourth Military Medical University, Xi’an, China; ^3^ Department of General Practice, Xijing Hospital, the Fourth Military Medical University, Xi’an, China

**Keywords:** HFpEF, inflammation, gut microbiota, metabolites, probiotics, fecal microbial transplantation

## Abstract

Heart failure with preserved left ventricular ejection fraction (HFpEF) is a disease that affects multiple organs throughout the body, accounting for over 50% of heart failure cases. HFpEF has a significant impact on individuals’ life expectancy and quality of life, but the exact pathogenesis remains unclear. Emerging evidence implicates low-grade systemic inflammation as a crucial role in the onset and progression of HFpEF. Gut microbiota dysregulation and associated metabolites alteration, including short-chain fatty acids, trimethylamine N-oxides, amino acids, and bile acids can exacerbate chronic systemic inflammatory responses and potentially contribute to HFpEF. In light of these findings, we propose the hypothesis of a “gut microbiota-inflammation-HFpEF axis”, positing that the interplay within this axis could be a crucial factor in the development and progression of HFpEF. This review focuses on the role of gut microbiota dysregulation-induced inflammation in HFpEF’s etiology. It explores the potential mechanisms linking dysregulation of the gut microbiota to cardiac dysfunction and evaluates the therapeutic potential of restoring gut microbiota balance in mitigating HFpEF severity. The objective is to offer novel insights and strategies for the management of HFpEF.

## 1 Introduction

Heart failure with preserved left ventricular ejection fraction (HFpEF) is a systemic multi-organ disease that often coexists with chronic metabolic conditions such as obesity, diabetes, and hyperlipidemia (Joseph et al., 2020; Hamo et al., 2024). HFpEF represents the most common form of heart failure (HF), comprising over 50% of HF cases (Nair, 2020). It is particularly prevalent among women aged 65 and older who frequently present with HF symptoms despite having a left ventricular ejection fraction exceeding 50% (Anselmi et al., 2021; Museedi et al., 2024). An Irish prospective study revealed that hospital admissions for non-cardiovascular emergencies or annual readmission was significantly higher for individuals with HFpEF compared to those with heart failure with reduced left ventricular ejection fraction (HFrEF) (Murphy et al., 2017). As the population ages, the prevalence, mortality, and economic burden of HFpEF are escalating, making it the most significant unmet medical need in cardiovascular diseases (Savarese et al., 2022), which cause a serious burden on society.

Studies have indicated that inflammation is a fundamental driver in the progression of HFpEF (Sanders-van-Wijk et al., 2020; Liu et al., 2024), promotes myocardial remodeling and dysfunction (Omote et al., 2022). It has been pointed out that during the development of HFpEF, the inflammatory pathway is distinguished from neurohumoral and metabolic pathways (Park et al., 2022) and may play a more significant role in HFpEF compared with HFrEF (Mongirdienė and Liobikas, 2022). Inflammatory states can activate coronary microvascular endothelial cells, inducing a reduction in nitric oxide (NO) bioavailability and impairment of the NO-cyclic guanosine monophosphate (cGMP)-protein kinase G (PKG) pathway in neighboring cardiomyocytes (Paulus and Tschöpe, 2013; Chirinos et al., 2016). Additionally, coronary inflammation stimulates the local release of transforming growth factor-beta (TGF-β), inducing fibroblast-to-myofibroblast differentiation and subsequent collagen secretion (Kapur, 2011; Antar et al., 2023), ultimately leading to myocardial diastolic dysfunction (Westermann et al., 2011).

**Figure 1:**
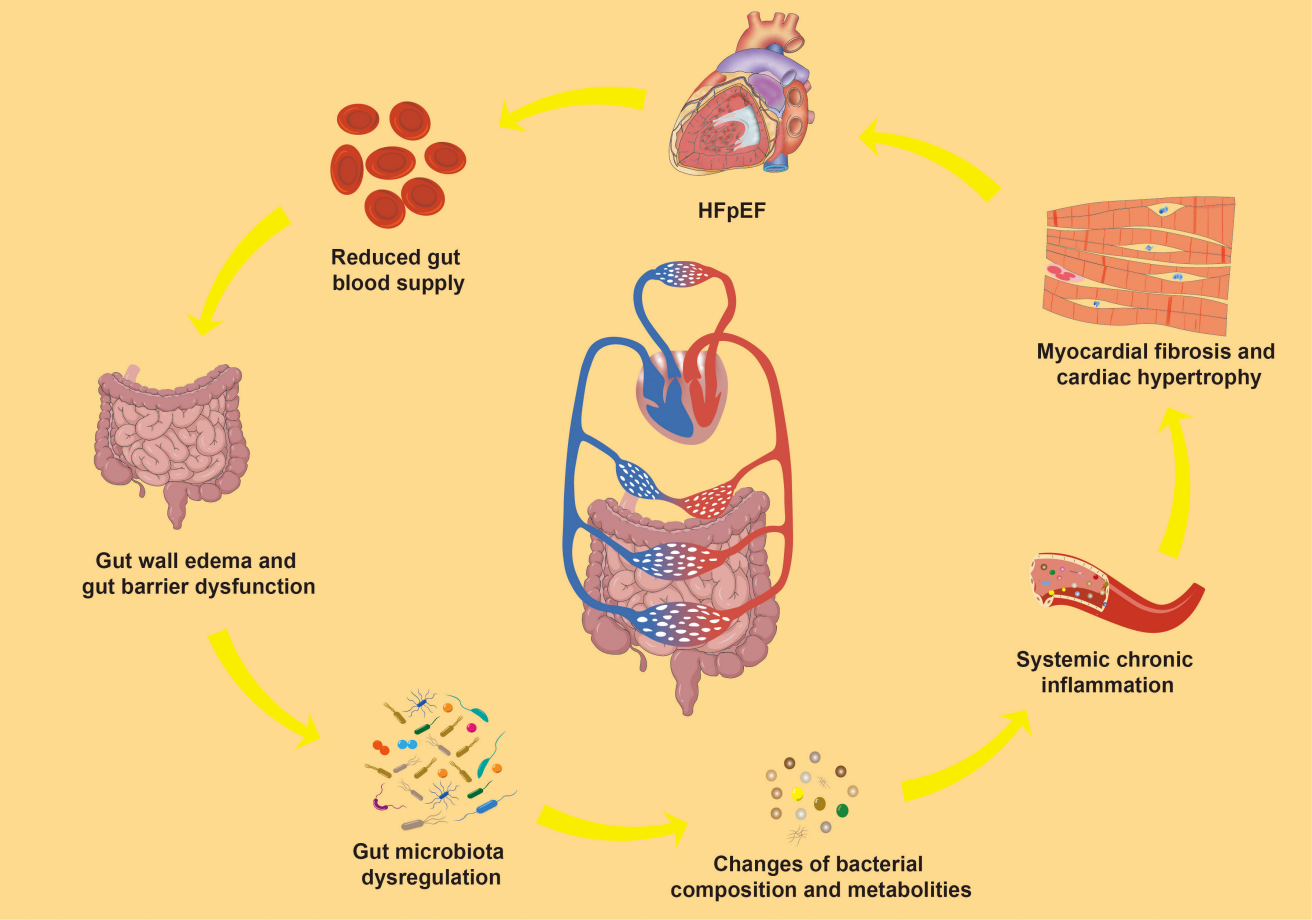
Gut microbiota-inflammation-HFpEF axis. HFpEF, Heart failure with preserved left ventricular ejection fraction.

Inflammation is closely associated with gut microbiota dysregulation. The gut microbiota, considered an “invisible organ” within the human body, participates in various physiological and metabolic processes, playing a crucial role in human health (Hou et al., 2022). In this condition, the gut microbiota significantly influences the inflammatory process through its metabolic activities and interactions with the immune system (Randeni et al., 2024). Dysregulation of the gut microbiota is common in individuals with HFpEF, manifesting as a decrease in beneficial microbes and an increase in potentially harmful bacteria (Wang et al., 2023; Caldarelli et al., 2024). The latest study found correlations between prealbumin, left ventricular ejection fraction, and NT-proBNP and multiple gut microbes in patients with HFpEF (Yang et al., 2024). This can be attributed to the disturbance of gut microbiota that compromises intestinal barrier function, allowing lipopolysaccharide (LPS), trimethylamine N-oxide (TMAO), and other harmful metabolites to enter the bloodstream and trigger systemic chronic inflammation, potentially leading to HFpEF (O’Donovan et al., 2020; Masenga et al., 2023). Conversely, HFpEF can reduce gut blood supply and induce changes in intestinal cell tight junctions, leading to intestinal wall edema and barrier dysfunction (Koufou et al., 2024), worsening gut microbiota dysregulation and creating a vicious cycle (Stolfi et al., 2022). This suggests a bidirectional communication axis between gut microbiota and HFpEF (**Figure 1**).

Inflammatory and gut microbiota have profound impacts on cardiovascular disease (Martins et al., 2024), and a robust association between gut microbiota and HFpEF has been established recently (O’Donovan et al., 2020), with HFpEF development likely linked to gut microbiota dysregulation-mediated immune inflammation (Perticone et al., 2024). Dapagliflozin’s positive effects on HFpEF may also be attributed to gut microbiota (Guan et al., 2023). However, the role of gut microbiota-induced inflammation in HFpEF remains unclear. Further in-depth investigation of the gut microbiota of HFpEF patients holds promise for effective prevention and treatment strategies (Mamic et al., 2023). In this review, we conducted a comprehensive search of the most relevant studies from PubMed, primarily focusing on English-language literature from all years. Some review articles and their reference lists were also searched to identify related articles. Search terms related to “heart failure with preserved ejection fraction”, “diastolic dysfunction”, “heart failure, diastolic”, “myocardial fibrosis”, “inflammation”, and the names of each specific metabolite produced by the gut microbiota were used to identify eligible studies. These studies analyzed the impact of inflammation triggered by gut microbiota imbalance on the HFpEF development or investigated the potential benefits of interventions aimed at correcting gut microbiota imbalances to alleviate HFpEF symptoms. By examining the complex interplay among gut microbiota dysregulation, inflammation and heart function, the goal is to uncover novel treatment strategies for managing HFpEF.

## 2 The unique pathophysiological mechanisms of HFpEF

Patients with HFpEF frequently suffer from multiple metabolic disorders, including obesity, hypertension, and diabetes (Mishra and Kass, 2021). Specifically, 84% of HFpEF patients are obese, over 60% have hypertension, and more than 20% have type 2 diabetes (Shah et al., 2016). Studies have demonstrated that obesity is a key factor contributing to impaired cardiac energy metabolism in HFpEF, and weight reduction has been shown to enhance myocardial glucose oxidation and improve cardiac function (Güven et al., 2024). Angiotensin II and aldosterone promote myocardial hypertrophy and fibrosis. While activation of the renin-angiotensin-aldosterone system (RAAS) is common in HFpEF patients, it is not a central pathophysiological mechanism (Liu et al., 2024). RAAS inhibitors can reduce cardiac hypertrophy, but do not significantly improve diastolic function, fibrosis, myocardial inflammation, endothelial activation, or oxidative stress (Telesca et al., 2024).

Concentric left ventricular hypertrophy is the most common structural myocardial abnormality in HFpEF, typically resulting from cardiomyocyte hypertrophy and myocardial interstitial fiber deposition. Unlike HFrEF characterized by centrifugal hypertrophy due to cardiomyocyte injury and activation of the neurohumoral system, systemic low-grade inflammatory response and oxidative stress play a more prominent role in left ventricular hypertrophy in HFpEF (Heinzel et al., 2015). Left ventricular diastolic dysfunction, marked by impaired relaxation, increased ventricular stiffness, and reduced elastic resilience, forms the pathophysiological basis of HFpEF (Obokata et al., 2020). Furthermore, systemic inflammation, elevated left ventricular filling pressure, abnormal left atrial structure and function, pulmonary hypertension, and right ventricular dysfunction collectively contribute to the unique pathophysiology of HFpEF.

## 3 The important role of inflammation in HFpEF

**Figure 2:**
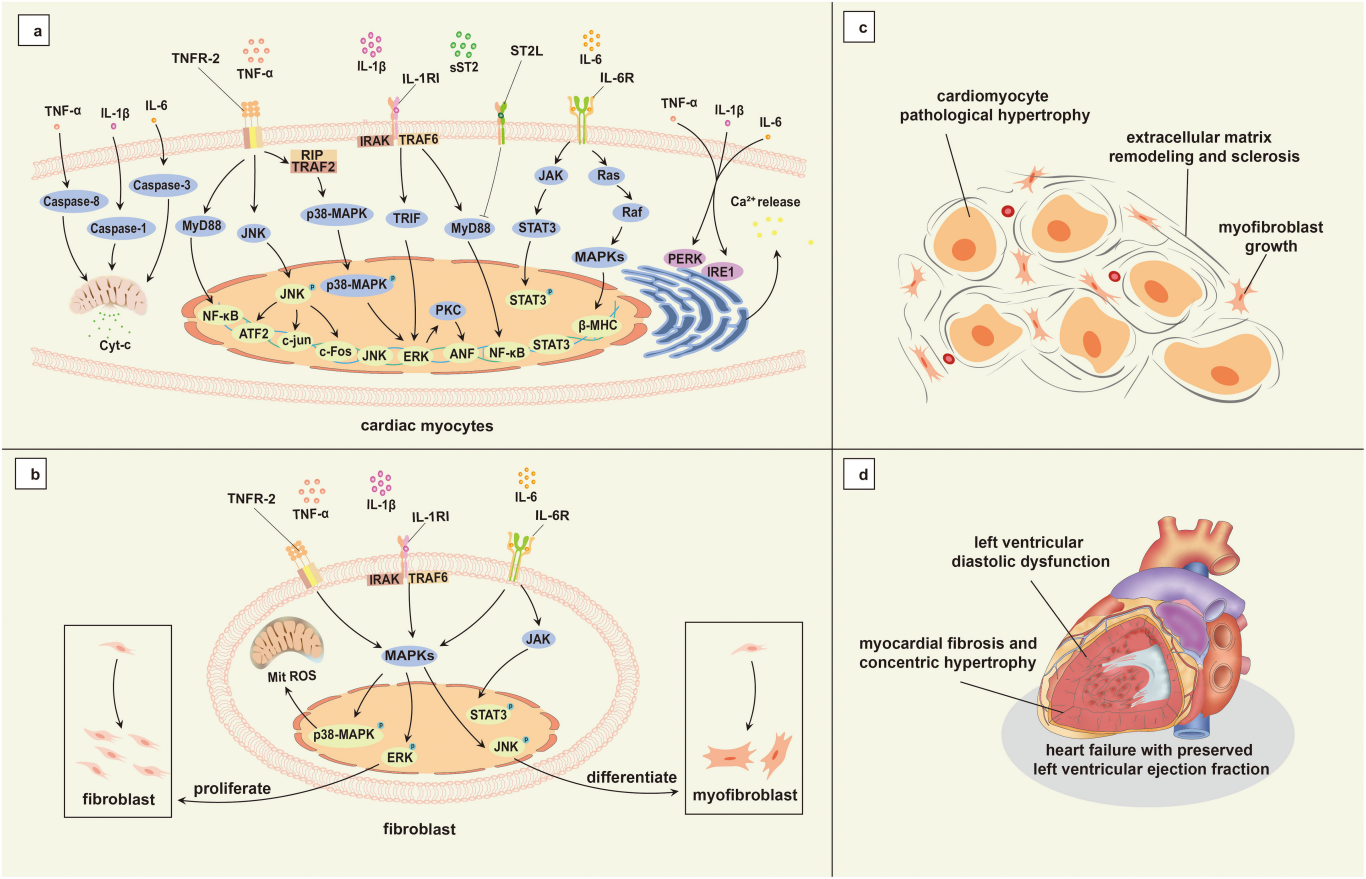
Mechanisms associated with inflammation in HFpEF. **(a)** Genes associated with cardiomyocyte hypertrophy induced by inflammatory cytokine activation; **(b)** Inflammatory factors activate fibroblasts and promote their proliferation and differentiation; **(c)** Cardiomyocyte hypertrophy and myocardial interstitial fibrosis; **(d)** Main pathophysiology of HFpEF. TNF-α, tumor necrosis factor-alpha; IL-1β, interleukin-1 beta; IL-6, interleukin-6; TNFR-2, tumor necrosis factor receptor-2; RIP, receptor-interacting protein; TRAF2, tumor necrosis factor receptor associated factor 2; MyD88, myeloid differentiation primary response 88; JNK, c-Jun N-terminal kinase; NF-κB, nuclear factor-kappa B; p38-MAPK, p38 mitogen-activated protein kinase; ATF-2, Activating Transcription Factor 2; c-Fos, cellular Fos; ERK, extracellular signal-regulated kinase; IL-1RI, type I interleukin-1 receptor; TRAF6, tumor necrosis factor receptor associated factor 6; IRAK, interleukin-1 receptors associated kinase; TRIF, toll/interleukin-1 receptor domain-containing adapter inducing interferon-beta; PKC, protein kinase C; ANF, atrial natriuretic factor; ST2L, transmembrane binding receptor suppression of tumorigenicity 2; sST2, soluble suppression of tumorigenicity 2; IL-6R, interleukin-6 receptor; JAK, janus kinase; STAT, signal transducer and activator of transcription; Ras, rat sarcoma; Raf, rapidly accelerated fibrosarcoma; MAPKs, mitogen-activated protein kinases; β-MHC, beta-myosin heavy chain; PERK, Protein Kinase RNA–like Endoplasmic Reticulum Kinase; IRE1, Inositol-Requiring Enzyme 1; HFpEF, heart failure with preserved left ventricular ejection fraction; Cyt-c, Cytochrome C; Mit, mitochondria; ROS, reactive oxygen species.

Numerous studies have reported that inflammatory markers, including tumor necrosis factor-alpha (TNF-α), C-reactive protein (CRP), interleukin-1 (IL-1), IL-6, and soluble suppression of tumorigenicity 2 protein (sST2) are significantly higher in individuals with HFpEF compared to those with HFrEF (Sanders-van-Wijk et al., 2015; Schiattarella et al., 2021; Dawuti et al., 2023). Additionally, inflammatory cells are markedly increased in myocardial biopsy samples from individuals with HFpEF (Westermann et al., 2011). Elevated levels of inflammatory factors may stimulate an inflammatory response in cardiac cells, leading to cardiomyocyte pathological hypertrophy, dysfunction, myofibroblast growth, extracellular matrix remodeling and sclerosis, and microvascular disease, which may promote abnormal myocardial remodeling, and ultimately cause HFpEF (Wenzl et al., 2021) (**Figure 2**).

IL-1 is a key regulator of inflammatory responses (Dinarello, 2011), IL-1β plays a major role in augmenting cardiac inflammation through its interaction with the IL-1 receptor, IL-1 receptor-associated kinase (IRAK), and tumor necrosis factor receptor-associated factor 6 (TRAF6). This interaction activates downstream signaling pathways, including nuclear factor kappa B (NF-κB), c-Jun N-terminal kinase (JNK)/activator protein 1 (AP-1), and mitogen-activated protein kinase (MAPK)/extracellular signal-regulated kinase (ERK) (Weber et al., 2010; Bent et al., 2018; Tang et al., 2024), all of which are implicated in cardiac injury. Consequently, IL-1 may contribute to cardiomyocyte hypertrophy, impaired function, and extracellular matrix accumulation through these activated pathways.

IL-6 activates the janus kinase (JAK)/signal transducer and activator of transcription (STAT), MAPK, and phosphoinositide 3-kinase (PI3K) pathways by binding to the IL-6 receptor and gp130, triggering intracellular signaling that induces inflammatory responses and can even lead to inflammatory storms (Rose-John, 2020). In cardiomyocytes, IL-6 downregulates sarcoplasmic reticulum Ca2+-ATPase expression, diminishes Ca2+ reuptake by the sarcoplasmic reticulum, and impacts diastolic relaxation (Wu et al., 2011).

SST2 serves as an inflammation marker and indicator of myocardial stress, and it dampens the cardioprotective effects of transmembrane binding receptor suppression of tumorigenicity 2 (ST2L) via the myeloid differentiation primary response 88 (MyD88)/IRAK/ERK/NF-κB signaling pathway by competing with IL-33 (Kakkar and Lee, 2008). SST2 is also recognized as a marker of inflammation and fibrosis. Studies have demonstrated a positive correlation between sST2 and left ventricular hypertrophy (Hubesch et al., 2022), and this correlation significantly enhances the prognostic value of HFpEF (Lebedev et al., 2020; Lotierzo et al., 2020; Yamamoto et al., 2021).

TNF-α, as a key proinflammatory factor, activates NF-κB and MAPK signaling pathways by binding to its receptors TNFR-1 and TNFR-2 (Liu et al., 1996; Bradley, 2008), aggravating cardiac inflammatory damage and promoting myocardial fibrosis and heart failure (Franssen et al., 2016). These pathways play a crucial role in the propagation of cardiac inflammation and the progression of HFpEF.

## 4 Gut microbiota and its metabolic changes in HFpEF

Individuals with HFpEF exhibit significant gut microbiota dysregulation compared to healthy individuals, characterized by reduced species richness, especially those with anti-inflammatory effects such as *Butyricicoccus*, *Sutterella*, *Lachnospira*, *Ruminiclostridium*, etc (Hummel et al., 2019). Concurrently, there is an observed increase in proinflammatory associated microbiota abundance like *Erysipelotrichaceae (* Drapkina et al., 2022). Regardless of the underlying etiology, gut microbiota dysregulation is consistent in HFpEF patients (Huang et al., 2021). Although the abundance of *Firmicutes*, *Bacteroides*, and *Proteus* reduced in HFpEF patients, these phyla continue to dominate the gut microbiota composition (Kaburova et al., 2020b). An Australian study corroborated these findings, emphasizing a significant depletion of short chain fatty acids (SCFAs) producing bacteria in HFpEF patients, particularly *Ruminiclostridium*. Importantly, this depletion is independent of confounding factors such as age, body mass index (BMI), and hypertension (Beale et al., 2021). The decrease in *Ruminiclostridium* may be linked to elevated levels of the increased N-terminal propeptide of procollagen type III, an indicator of myocardial fibrosis (Kaburova et al., 2021). Details are shown in **Table 1**.

**Table 1:** Gut microbiota changes in the HFpEF.

Sample characteristics	Methods	Gut microbiome	Other key findings	Conclusion
26 HFpEF vs 30 healthy controls, Healthy controls were age-matched with HFpEF (Hummel et al., 2019).	16S rRNA	The abundance of species such as *Butyricicoccus*, *Sutterella*, *Lachnospira*, *Ruminiclostridium* is reduced in patients with HFpEF compared with controls.	24 OTUs were differentially present between HFpEF and healthy controls, with *Prevotella* abundance the strongest differentiator.	The gut microbiome differs between HFpEF and age-matched healthy controls
59 HFpEF vs 50 controls. Three different methods were used to detect intestinal flora in the three categories of people with HFpEF, HFrEF, ASCVD and compare with healthy controls (Drapkina et al., 2022)	MALDI-TOF-MS, NGS, qPCR	MALDI-TOF-MS analysis shows reduced abundance of *Enterococcus faecium*, *Enterococcus faecalis*, *Bacteroides fragilis* in patients with HFpEF. 16S rRNA sequencing analysis reveals increased abundance of the *Erysipelotrichaceae* in patients with HFpEF. Patients with HFpEF has higher abundance of *Oscillibacter* sp. by qPCR.	Patients with HFpEF have minimal difference compared with controls compared with patients with ASCVD and HFrEF. The study did not find a lower abundance of *Butyricicoccus*, *Sutterella*, *Lachnospira*, and *Ruminiclostridium* in the HFpEF group than in the control group.	Differences in gut microbiome between patients with cardiovascular disease and healthy persons. The differences in the conclusions drawn by the three methods require a comprehensive approach to the microbiota.
30 HFpEF vs 30 controls (Huang et al., 2021)	Illumina high-throughput DNA sequencing	At the phylum classification level, the abundance of *Synergistetes* tends to be higher in the HFpEF group At the genus classification level, the abundance of *Butyricicoccus*, *Sutterella*, *Lachnospira*, and *Ruminiclostridium* in the HFpEF group are decreased, the abundance of *Enterococcus* and *Lactobacillus* are *increased*.	The species richness of gut microbiota in the HFpEF group is decreased. Differences in composition and species diversity of gut microbiota in patients with HFpEF due to different etiologies.	Patients with HFpEF have an increased abundance of microbiota associated with inflammation and a decreased abundance of microbiota associated with anti-inflammatory effects in the gut environment. The species richness of gut microbiota in HFpEF patients tends to be lower.
42 HFpEF (Kaburova et al., 2020b)	16S rRNA	The relative abundance of the most prevalent phyla in gut microbiota is *Firmicutes*, *Bacteroidetes* and *Proteobacteria*.	ECV significant positively correlated with *Faecalibacterium*, *Blautia*, *Lachnoclostridiu*. ECV positively correlated with *Holdemania*, *Victivallis*, *Dehalobacterium*, *Enterococcus* and *Catabacter*.	Both negative and positive significant correlations between marker of myocardial fibrosis and several bacterial genera
26 HFpEF vs 67 controls. All patients underwent invasive exercise hemodynamics (Beale et al., 2021)	16S rRNA	The ratio of *Firmicutes* to *Bacteroidetes* tends to be lower in patients with HFpEF compared with controls(doesn’t reach statistical significance). Patients with HFpEF has a significant depletion of bacteria known to be SCFA producers, particularly *Ruminococcus*.	The depletion of *Ruminococcus* in patients with HFpEF appears independent of BMI, age, and hypertension. β‐diversity analysis different between HFpEF and controls independent of age, sex, BMI, systolic blood pressure, dietary quality, and fiber intake, suggesting that the changes seen in HFpEF are far beyond that explained by this factors.	Significant differences in gut microbiota between patients with HFpEF and controls with differences in abundance of microbial classification groups. The gut microbiota and its metabolites, particularly SCFAs, may be targets for future prevention and possibly treatment of HFpEF.
42 HFpEF (Kaburova et al., 2021)	16S rRNA	The highest abundance in patients with HFpEF is *Firmicutes*, *Bacteroides*, and *Proteobacteria*.	*Allisonella* associated with higher PICP concentrations, *S. ruminalis* and *Gemmiger* associated with lower PICP levels, *Blautia* and the unclassified *Enterobacteriaceae* associated with higher PIIINP levels, and *Bilophila* associated with lower PIIINP levels	The gut microbiota may be associated with myocardial fibrosis generation based on pathways mediated by SCFA, histamine, and proinflammatory compounds.
44 HFpEF vs 45 healthy controls (Kaburova et al., 2019)	16S rRNA	*Bacteroides*, *Alistipes*, *Pseudomonas*, and *Fusobacterium* are enriched in the common core microbiota of HFpEF patients, while *Lachnospira*, *Roseburia*, *Eubacterium*, *Methanobrevibacter*, *Faecalibacterium*, *Lactobacillus* and *Bifidobacterium* are depleted.	The patients with HFpEF has significantly higher levels of the TMAO.	Significant structural alterations of the intestinal microbiome and increased TMAO levels in HFpEF patients’ serum.
42 HFpEF (Kaburova et al., 2020a)	16S rRNA	–	Significant correlations between PICP and the *Ruminococcus*, *Gemmiger*, *Allisonella* and *Howardella*. PIIINP significantly correlated with *Blautia* and *Bilophila*.	Both PICP and PIIINP has negative significant correlations with beneficial bacterial genera and positive correlations with several potentially harmful gut bacterial genera.

HFpEF, heart failure with preserved left ventricular ejection fraction; OTUs, operational taxonomic units; SCFAs, short-chain fatty acids; TMAO, trimethylamine oxide; HFrEF, heart failure with reduced left ventricular ejection fraction; ASCVD, atherosclerotic cardiovascular disease; MALDI-TOF-MS, matrix-assisted laser desorption ionization time of flight mass spectrometry; NGS, next generation sequencing; qPCR, quantitative polymerase chain reaction; ECV, extracellular volume; BMI, body mass index; PICP, procollagen I carboxy terminal Propeptide; PIIINP, procollagen III N-terminal peptide.

Alterations in the gut microbiota are associated with alterations in their metabolites. In individuals with HFpEF, these changes are characterized by an increase in harmful products synthesis and a decrease in beneficial metabolite levels. The reduction in the SCFA-producing microbiota in individuals with HFpEF affects SCFAs synthesis (Beale et al., 2021). A cross-sectional study involving 324 participants revealed significantly elevated TMAO levels in individuals with HFpEF compared to healthy controls (Guo et al., 2020), and alterations in gut microbiota composition identified as a primary driver of increased TMAO levels (Martin et al., 2008). Gut microbiota dysregulation in individuals with HFpEF can also trigger changes in the metabolites of dietary amino acids (AAs) within the gut, such as increased indoxyl sulfate, a byproduct of tryptophan metabolism, and decreased production of protective indole-3-propionate (Mamic et al., 2021). Meanwhile, gut microbiota in individuals with HFpEF play a crucial role in all stages of bile acids (BAs) metabolism. The microbiota can indirectly contribute to BAs synthesis by regulating BAs hydrolase activity, once BAs enter the intestine, the microbiota facilitates their further metabolism into various types (Shi M. et al., 2023). Briefly, gut microbiota dysregulation can lead to alterations in metabolic processes, thereby inducing a cascade of physiological changes.

## 5 Gut microbiota -inflammation-HFpEF axis

With the in-depth investigation of the relationship of gut microbiota to cardiovascular disease, it has become evident that gut microbiota dysregulation is an important contributor to inflammatory stimulation, exerting considerable effects on cardiac and vascular health (Petruzziello et al., 2024). This dysregulation can induce both local and systemic inflammation, which is considered a critical potential mechanism for the onset of HF (Hanna and Frangogiannis, 2020). The resulting low-grade chronic inflammation stimulates monocytes, endothelial cells and other cells, prompting them to secrete pro-inflammatory cytokines (Violi et al., 2023), including TNF-α, IL-1β, IL-6, cellular adhesion molecules (CAMs), etc. These cytokines can induce chronic cardiac fibrosis, which may contribute to the development of HFpEF (Hanna and Frangogiannis, 2020; Thomas and Grisanti, 2020). In recent years, a variety of evidence has suggested that inflammation induced by pattern recognition receptors associated with bacterial, as well as their metabolites, including SCFAs (Challa and Lewandowski, 2022), TMAOs (Kinugasa et al., 2021), AAs (Wang et al., 2024), BAs (Shi M. et al., 2023) are involved in the development of HFpEF (**Table 2**). Some of these bacterial metabolites are being considered as potential prognostic markers for HFpEF (Modrego et al., 2023). This may be due to the fact that these metabolites affect the NF-κB signaling pathway by different pathways, ultimately affecting the activation of Nod-like receptor pyro-protein domain-associated protein 3 (NLRP3) inflammasomes.

**Table 2:** The effect of gut microbiota and its metabolites on the pathophysiology of HFpEF.

Factors	Model	Downstream signaling	Phenotype
LPS (Singh et al., 2012)	C57BL/6J mice	TLR4/MyD88/CaMKII	Induced cardiomyocyte hypertrophy and inflammation
LPS (Zhu et al., 2024)	C57BL/6J male mice	TLR4/MyD88	Decreasing cardiomyocyte hypertrophy and interstitial fibrosis
Fibrinogen (Li et al., 2009)	SD rats	TLR4/MyD88/NF-κB	Cardiac hypertrophy
Flagellin (Liu et al., 2015)	C57BL/6J mice	TLR5	Interstitial cardiac fibrosis and dysfunction
SCFAs (Kaye et al., 2020)	C57BL/6J mice	GPR43 and GPR109A	Decreased cardiac hypertrophy and cardiac fibrosis
SCFAs (Lin et al., 2022)	ABX mice	GPR41 and GPR43/SMAD2/TGF-β1	Restored cardiac function and prevented excessive fibrosis and ECM disarray under stress
SCFAs (Dong et al., 2024)	Mouse CFs (FH-M187)	NLRP3/Caspase-1/TGF-β1	Repressed the transdifferentiation of CFs into MFs
TMAO (Li et al., 2019)	SD rats	TGF-β/SMAD3	Cardiac hypertrophy and fibrosis
TMAO (Wang et al., 2020)	C57BL/6J	p65 NF-κb TGF-β/SMAD3	Cardiac hypertrophy and fibrosis
TMAO (Wang et al., 2024)	SD rats	PKC/NF-κB	Inducing inflammatory responses, myocardial hypertrophy and fibrosis
Tryp, Kyn (Carrillo-Salinas et al., 2020)	C57BL/6J male mice	AhR	Slow down the development of adverse cardiac remodeling
Kyn (Shi B. et al., 2023)	Patients and mice model	AhR–targeted genes	Aggravates cardiac remodeling, hypertrophy and fibrosis
Urolithin A (Chen et al., 2022)	SD rats	TGF-β1/Nrf2	Inhibits myocardial fibrosis
Cholic acid (Eblimit et al., 2018)	C57BL/6J male mice	TGR5/Akt/PKA/ERK	Attenuate cardiac hypertrophy
TUDCA (Rani et al., 2017)	Male mice	Downregulated GRP78 and GRP94. Decreased the phosphorylation of PERK and eIF2α	Reduced myocardial hypertrophy, attenuated cardiac fibrosis
TUDCA (Turdi et al., 2013)	C57BL/6J male mice	Upregulated SERCA2a expression	Attenuated diastolic dysfunction

LPS, lipopolysaccharide; SCFAs, short-chain fatty acids; TMAO, trimethylamine oxide; Trp, tryptophan; Kyn, kynurenine; TUDCA, tauroursodeoxycholic acid; ABX, depleted of gut microbiota with antibiotics; ECM, extracellular matrix; CFs, cardiac fibroblasts; MFs, myofibroblasts; eIF2α, eukaryotic translation initiation factor 2α; SERCA2a, sarcoplasmic/endoplasmic reticulum calcium atpase 2a. TLR,toll-like receptors; MyD88, myeloid differentiation primary response 88; CaMKII, Calcium/calmodulin-dependent protein kinase II; NF-κB, nuclear factor-kappa B; GPR, G protein-coupled receptors; SMAD, small mother against decapentaplegic family member; TGFβ, TGF-β, transforming growth factor-β; NLRP-3, nucleotide-binding oligomerization domain-like receptor family pyrin domain containing 3; PKC, protein kinase C; AhR, aryl hydrocarbon receptor; Nfr, nuclear factor erythroid 2-related factor; Akt, protein kinase B; PKA, protein kinase A; ERK, extracellular signal-regulated kinase.

**Figure 3:**
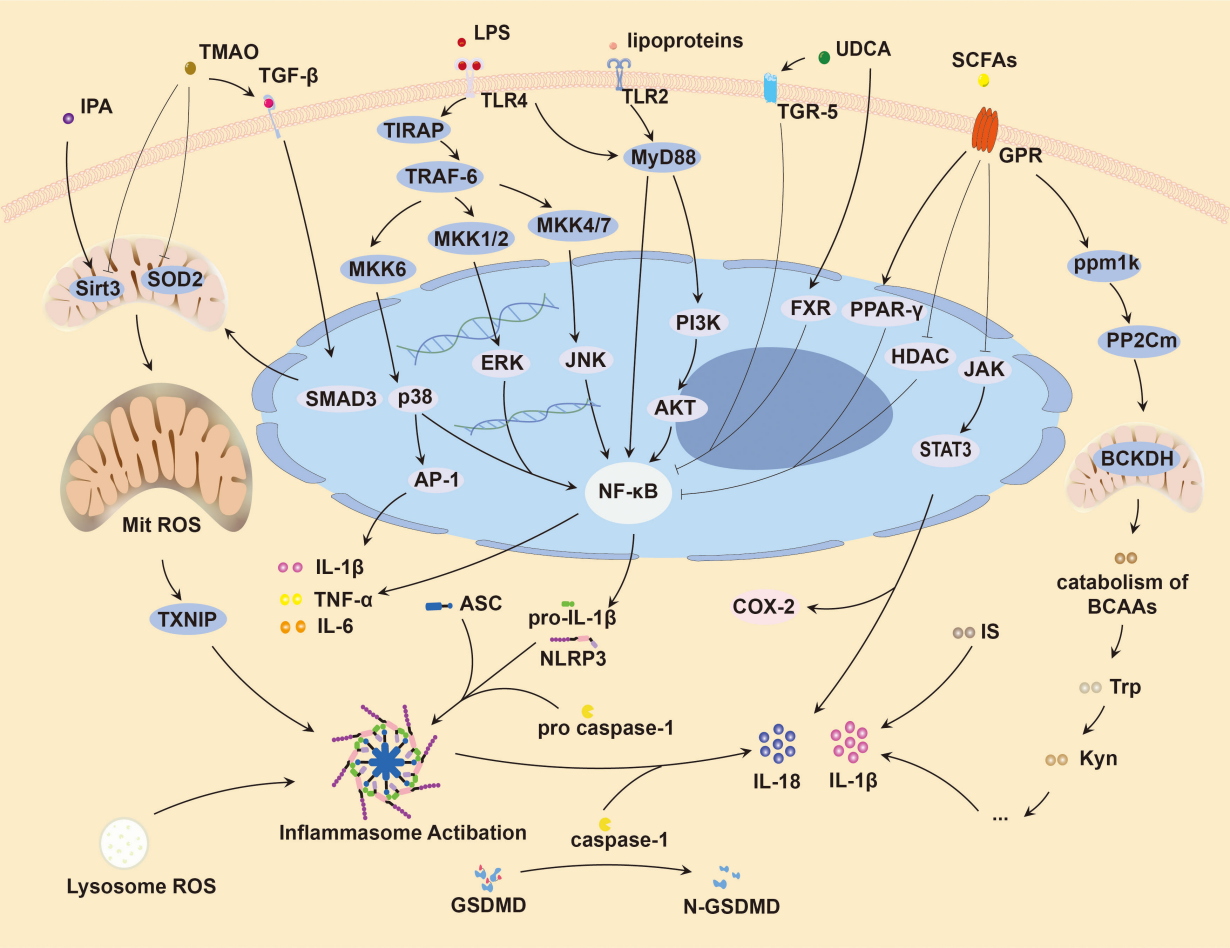
Inflammation induced by gut microbiota causing HFpEF. HFpEF, Heart failure with preserved left ventricular ejection fraction; LPS, lipopolysaccharide; TMAO, trimethylamine oxide; SCFAs, short-chain fatty acids; IPA, indole-3-propionic acid; Trp, tryptophan; Kyn, kynurenine; IS, indole sulfate; BCAAs, branched chain amino acids; UDCA, ursodeoxycholic acid; TLR, toll-like receptor; Mit, mitochondrion; SOD2, superoxide dismutase 2; ROS, reactive oxygen species; COX-2, cyclooxygenase-2; TGF-β, transforming growth factor-β; TGR-5, transmembrane G protein-coupled receptor; FXR, farnesoid X receptor; PPAR-γ, peroxisome proliferator-activated receptor-γ; GPR, G protein-coupled receptors; HDAC, histone deacetylase; NLRP3, nucleotide-binding oligomerization domain-like receptor family pyrin domain containing 3; ASC, apoptosis-associated speck-like protein containing a caspase activation and recruitment domain; SIRT3, sirtuin 3; BCKDH, branched-chain α-keto acid dehydrogenase; TNF-α, tumor necrosis factor α; IL, interleukin; TXNIP, thioredoxin-interactive protein; GSDMD, gasdermin D; GSDMD-N, N-terminal gasdermin D; Smad3, small mother against decapentaplegic family member 3; TIRAP, toll/interleukin-1 receptor domain-containing adapter protein; TRAF-6, tumor necrosis factor receptor associated factor 6; MKK6, mitogen-activated protein kinase kinase 6; AP-1, activator protein-1; MAPKs, mitogen-activated protein kinases; JNK, c-Jun N-terminal kinase; ERK, extracellular signal-regulated kinase; NF-κB, nuclear factor-kappa B; MyD88, myeloid differentiation primary response 88; PI3K, Phosphoinositide 3-kinase; AKT, protein kinase B; JAK, janus kinase; STAT3, signal transducer and activator of transcription 3; ppm1k, protein phosphatase Mg2+/Mn2+-dependent 1K; PP2Cm, protein phosphatase 2Cm.

Emerging evidence suggests that innate immune response pathways from NLRP3 inflammasomes to IL-1, IL-6 play an important role in HFpEF (Li et al., 2022; Cheng et al., 2023). Activation of the NLRP3 inflammasome mediated by the NF-κB signaling pathway (Bauernfeind et al., 2009), upregulates transcription of NLRP3, pro-IL-1β, IL-18 (Grebe et al., 2018; Olsen et al., 2021). The NLRP3 protein interacts with and binds to apoptosis-associated speck-like protein containing a caspase activation and recruitment domain, which subsequently recruits pro-caspase-1, facilitating its maturation and activation. This cascade leads to cleavage and release of pro-IL-1β and IL-18, inducing vascular endothelium and smooth muscle cells to produce and release large quantities of IL-6 (Libby, 2021). In experimental models of HFpEF, NLRP3 inflammasome activation and increased expression of IL-1β and IL-18 have been found in the hearts of affected mice (Yang et al., 2020; Deng et al., 2021). Furthermore, NLRP3 inflammasomes are implicated in induction of ventricular arrhythmias in HFpEF mice, contributing to a poor prognosis (Yang et al., 2020). Notably, inhibition of NLRP3 inflammasomes has been shown to reduce cardiac inflammation (Zhao et al., 2021; Yang et al., 2024) and improve left ventricular diastolic dysfunction and myocardial fibrosis in patients with HFpEF (Cheng et al., 2023). Potential mechanisms by which gut microbiota dysregulation may affect HFpEF through different inflammation pathways will be elaborated next (**Figure 3**).

### 5.1 Toll-like receptors pathway

TLRs are the key components of the pattern recognition receptor family, which significantly influence the innate immune system by recognizing pathogen-associated molecular patterns and injury-associated molecular patterns. So far, 10 different Toll-like receptors (TLRs, including TLR1-10) have been identified in humans, each exhibiting specificity for various microbial components. For instance, TLR2, as a dimeric cell membrane protein, is responsible for the recognition of bacterial lipoproteins and peptidoglycans. TLR4 is known for its recognition of LPS, while TLR5 is sensitive to bacterial flagella. Intracellular TLR3 detects double-stranded RNA, and TLR9 identifies bacterial DNA containing unmethylated CpG sequences (Bezhaeva et al., 2022).

In the context of gut microbiota dysregulation, LPS is secreted by Gram-negative bacteria, engages with a range of immune receptors and proteins, including TLR4, LPS-binding protein, and CD14 (Ryu et al., 2017). This interaction triggers TLR4 activation on the intestinal epithelium, leading to disruption of tight junctions between intestinal epithelial cells (Park and Lee, 2013; Mohr et al., 2022), increased intestinal permeability (Hietbrink et al., 2009), and facilitation of bacteria and endotoxins into the bloodstream. Once in the systemic circulation, LPS and peptidoglycans provoke an immune response by binding to TLR4 and nucleotide-binding oligomerization domain-like receptors (NLRs) (Rodrigues-e-Lacerda et al., 2023). Consequently, LPS is identified as a pivotal factor in induction of systemic inflammation (Cao et al., 2018).

The microbial constituents originating from the gut microbiota that can enter the bloodstream and reach the heart, inducing inflammation through activation of TLRs. This inflammatory cascade can ultimately lead to myocardial dysfunction and fibrosis (Zhang et al., 2023). Studies have demonstrated that TLRs, which orchestrate innate immune responses, are primarily responsible for inflammatory responses in the hearts of HF patients (Sharma et al., 2016), including TLR2, TLR3, TLR4, TLR5, and TLR9 (Yu and Feng, 2018). Specifically, TLR2 has been shown to mediate cardiac hypertrophy and inflammation in mice induced by Ang II via the TLR2/MyD88/NF-κB signaling pathway (Ye et al., 2021). Antagonizing TLR2 can block activation of NF-κB, suppress expression of inflammatory cytokines such as IL-1β, IL-6, IL-18, and attenuate cardiomyocyte apoptosis and cardiac fibrosis (Wang et al., 2019). TLR4 is implicated in cardiac inflammation and development of hypertrophy through multiple signaling pathways, including TLR4/MyD88/Calcium/calmodulin-dependent protein kinase II (CaMK II), TLR4/MyD88/NF-κB, TLR4/MyD88/PI3K/protein kinase B (AKT) and TLR4/MyD88/MAPK (Zhang et al., 2023). Animal experiments have found that *Lactobacillus reuteri* GMNL-263 can reduce inflammation, hypertrophy, and fibrosis in myocardial tissue by inhibiting theTLR4 pathway (Chiang et al., 2021). Both TLR3 and TLR4 have been identified to facilitate Ang II-induced cardiac hypertrophy through the TRIF pathway (Han et al., 2018). Furthermore, TLR5 has been linked to cardiac fibrosis and dysfunction by stimulating inflammation and endothelial-mesenchymal transition (Liu et al., 2015). Myocardial cell hypertrophy and fibrosis have been identified as the major pathophysiological manifestations of HFpEF (Fayyaz et al., 2024; Krittanawong et al., 2024).

### 5.2 Short chain fatty acids

SCFAs, primarily acetate, propionate, and butyrate, are critical mediators in the “gut microbiota-inflammation-HFpEF axis” and are terminal products of gut microbial catabolism (Yuan et al., 2024). Within the digestive tract, SCFAs can increase expression of intestinal tight junction proteins (Wang et al., 2012), regulate Treg/Th17 balance via peroxisome proliferator-activated receptor-gamma (PPAR-γ) pathway, and maintain gut epithelial cell physiology (Wen et al., 2021), thereby reducing gut permeability and alleviating low-grade systemic inflammation (Ma et al., 2022).

Butyrate reduction is prevalent in cardiovascular disease and associated with disease severity (Chakaroun et al., 2023). This is mainly because SCFAs are known to influence the pathology of HF by activating multiple G protein-coupled receptors, inhibiting histone deacetylase (HDAC) activity, and improving the cardiac inflammatory response (Kaye et al., 2020; Yukino-Iwashita et al., 2022; Taslim et al., 2023). Specifically, propionate diminishes the activity of NF-κB, IL-6, STAT1, and STAT3 signal transduction through down-regulation of HDAC activity, leading to a decrease in Th1 and Th17 immunoreactivity (Han et al., 2024). Additionally, propionate suppresses downstream inflammatory responses mediated by STAT1 and STAT3 (Jeong and Choe, 2023). Butyrate also significantly inhibits NK-κB activity by stimulating PPAR-γ (Malesza et al., 2021). Oral administration of propionate to mice with myocardial injury has been shown to substantially reduce systemic inflammation and myocardial fibrosis (Tang et al., 2019). In obesity mice, butyrate supplementation has been observed to suppress the expression of inflammatory factors such as IL-1β, NLRP3 and monocyte chemoattractant protein-1 in cardiac tissues. This effect is achieved by inhibiting HDAC and negatively regulating the NLRP3 inflammasome signaling pathway, which in turn reduces the release of inflammatory factors within adipocytes (Wang et al., 2015). Given the strong association between obesity, NLRP3 inflammasomes, and the progression of HFpEF, these findings are significant. Hatahet et al. treated obese pre-HFpEF mice with tributyrin, the transcript levels protein phosphatase Mg2+/Mn2+ dependent 1K (ppm1k) were significantly increased. Ppm1k encodes protein phosphatase 2Cm, which in turn causes dephosphorylation and activation of the branched-chain alpha-keto acid dehydrogenase complex, promoting branched-chain amino acids (BCAAs) catabolism and alleviating the early cardiac mechanical dysfunction associated with the development of obesity-related HFpEF (Hatahet et al., 2023).

Collectively, these studies suggest that SCFAs play a pivotal role in preventing the development of HFpEF by cutting off the connection between inflammation and the metabolic syndrome, suggesting their significant potential in the prevention and management of HFpEF (Beale et al., 2021; Challa and Lewandowski, 2022).

### 5.3 Trimethylamine N-oxide

TMAO is the oxygenated metabolite of trimethylamine, generated by gut microbiota catabolism of choline and L-carnitine, followed by hepatic enzymatic oxidation (Janeiro et al., 2018). *In vitro* screening tests indicate that 36 strains from four phyla, including *Bacteroidetes*, *Firmicutes*, *Actinobacteria*, and *Proteobacteria*, participate in these processes under anaerobic conditions (Romano et al., 2015). Studies have shown that TMAO levels significantly decrease with a diet cessation, and this is associated with reduced cardiac fibrosis (Organ et al., 2020).

TMAO has been implicated in cardiac dysfunction through multiple mechanisms (Makrecka-Kuka et al., 2017; Tang et al., 2019). Several studies have found that TMAO promotes myocardial hypertrophy and fibrosis via the TGF-β/SMAD3 signaling pathway (Li et al., 2019; Wen et al., 2022). Elevated plasma TMAO levels have been associated with cardiac inflammation and interstitial fibrosis, contributing to cardiac dysfunction in mice that consume a diet rich in choline (Chen et al., 2017). Administration of a TMAO synthesis inhibitor has shown the capacity to modulate the TGF-β/SMAD3 signaling pathway, thereby preventing myocardial hypertrophy and fibrosis (Wang et al., 2020). Other studies have revealed that TMAO inhibits sirtuin3 expression and suppresses the activity of superoxide dismutase 2 and mitochondrial aldehyde dehydrogenase 2, leading to mitochondrial reactive oxygen species accumulation and activation of NLRP3 inflammasomes and N-terminal gasdermin D production, which in turn contributes to cardiovascular endotheliitis (Sun et al., 2016; Zhang et al., 2020; Li et al., 2022). Additionally, TMAO stimulates the secretion of exosomes by hepatocytes, which activate the NF-κB signaling pathway, causing vascular endothelial diastolic dysfunction (Liu et al., 2021). In cardiomyocytes, modulation of the TMAO/NF-κB pathway inhibits myocardial hypertrophy and fibrosis, thereby ameliorating cardiac dysfunction (Wang et al., 2024). TMAO is also excreted by the kidney, and elevated levels can lead to renal interstitial fibrosis and dysfunction (Sun et al., 2017), indirectly promoting the development of HFpEF (Ter Maaten et al., 2016).

Previous studies have reported elevated plasma TMAO concentrations are associated with left ventricular diastolic dysfunction (Wilson Tang et al., 2015). While the prognostic value of TMAO levels in HF has been a subject of debated, with some suggesting its relevance is primarily for HFrEF, and not HFpEF (Wilson Tang et al., 2014; Trøseid et al., 2015; Suzuki et al., 2019). Subsequent studies have challenged this view. Notably, Salzano et al. have demonstrated elevated TMAO is associated with outcome in patients with HFpEF, especially when B-type natriuretic peptide levels are not elevated, suggesting its TMAO as a sensitive risk stratification marker for HFpEF (Salzano et al., 2020). Further studies have confirmed TMAO as an independent risk factor for HFpEF, and highly associated with HFpEF risk (Dong et al., 2021). In addition, plasma TMAO levels at discharge in individuals with HFpEF have been link to an increased risk of subsequent cardiovascular events post-discharge (Kinugasa et al., 2021).

### 5.4 Amino acids

Dysregulation of gut microbiota can affect the metabolism of AAs, increasing the risk of HF (Mahenthiran et al., 2024). Multiple metabolites derived from tryptophan have been shown to affect cardiac function. For instance, indole sulfate may increase the expression of pro-inflammatory cytokines, such as TNF-α, IL-1β in cardiomyocytes. This upregulation is mediated through activation of signaling pathways, including p38 MAPK, p42/44MAPK, and NF-κB, inducing myocardial fibrosis and hypertrophy, ultimately contributing to myocardial diastolic dysfunction (Lekawanvijit et al., 2010; Shimazu et al., 2013). In a recent study by Wang et al., exogenous supplementation of another Trp metabolite, indole-3-propionic acid supplementation reduced inflammation, oxidative stress, diastolic dysfunction, and myocardial remodeling in HFpEF. This beneficial effect is thought to be mediated by inhibition of nicotinamide N-methyltransferase and activation of sirtuin 3 (Wang et al., 2024).

Kynurenine is an important metabolite of AAs, has been positively associated with symptom severity, inflammation indicators, and mortality risk in patients with HF (Lund et al., 2020; Razquin et al., 2021). This may be due to kynurenic acid induction of pro-inflammatory IL-1β and IL-8 and the expression of TNF via the aromatic hydrocarbon receptor signaling (Dahlem et al., 2020). On the contrary, elevated inflammatory mediators can also mediate kynurenine pathway activation through induction of JAK-STAT signaling cascade (Grishanova and Perepechaeva, 2024). Other studies have found that circulating product concentrations resulting from gut microbiota metabolism of phenylalanine and tyrosine are associated with major cardiovascular events and mortality risk (Nemet et al., 2023). The accumulation of BCAAs and their intermediary metabolites in the myocardium is also associated with the development of HF (McGarrah and White, 2023). A BCAA-free diet attenuates the proliferation of cardiac fibroblasts and the expression of collagen 1a1, thereby mitigating cardiac hypertrophy induced by workload (Yang et al., 2023). Although dietary intake is the primary source of BCAAs, these indispensable amino acids are also synthesized by the gut microbiota (Gojda and Cahova, 2021). Although studies have established the effect of amino acids on the heart, further research is warranted to confirm the role of gut microbiota-mediated AAs metabolism in the pathogenesis of HFpEF.

### 5.5 Bile acids

BAs are organic acids synthesized by the liver and released into the small intestine, where they promote the absorption of dietary fat-soluble molecules (Wan et al., 2020). Bacteroides, Clostridium, Bifidobacterium, Lactobacillus, and other genera modulate the expression of bile salt hydrolase genes. Elevated levels of total and unconjugated BAs correlate with an increased abundance of the associated intestinal microbiota. Besides, the concentration of secondary BAs, including deoxycholic acid, is positively correlated with the relative abundance of Bacteroides (Yokota et al., 2012). This association suggests a significant interaction between the gut microbiota and BAs.

Recent research suggests that BAs act as signaling molecules mediating cardiac function (Yntema et al., 2023). BAs are divided into two categories based on molecular structure: hydrophilic and hydrophobic. These distinct types of BAs have differential effects on cardiac health. Hydrophobic BAs are associated with QT interval prolongation, cardiac hypertrophy, cardiomyocyte apoptosis, and dysregulation of cardiac hemodynamics (Vasavan et al., 2018).

Conversely, hydrophilic BAs, acting as steroidal signaling molecules, confer cardiac protection by modulating cardiac inflammation through several pathways. These include activation of the nuclear receptor FXR and the membrane G protein-coupled receptor 5 (TGR-5) in the myocardium, and the inhibition of NF-κB signaling (Xiaoli et al., 2020; Zhou and Anakk, 2022; Shi M. et al., 2023; Zhang et al., 2023). Activation of TGR-5 activates the AKT pathway in downstream cardiomyocytes, which reduces cardiomyocyte inflammation and oxidative stress and increases cardiomyocyte survival (Eblimit et al., 2018). Treatment with taurine deoxycholic acid attenuated hypertensive-induced cardiac inflammation and myocardial remodeling in animal experiments (Bal et al., 2019). In cell culture studies, activation of TGR-5 by lithocholic acid ameliorates hyperglycemia-induced cardiac hypertrophy in cardiomyocytes (Cheng et al., 2019). Vitamin D receptors (VDRs) are expressed within the T tubules of cardiomyocytes, with lithocholic acid (LCA) being one of its ligands (Tishkoff et al., 2008). Targeted ablation of VDR in cardiomyocytes leads to cellular hypertrophy, cardiac enlargement, and impairments in both systolic and diastolic function (Chen et al., 2011). However, it remains to be determined whether LCA ligands can modulate VDR to elicit similar effects. These studies revealed an important role for BAs in the pathophysiology of HFpEF.

## 6 Modulating gut microbiota dysregulation may improve HFpEF

**Figure 4:**
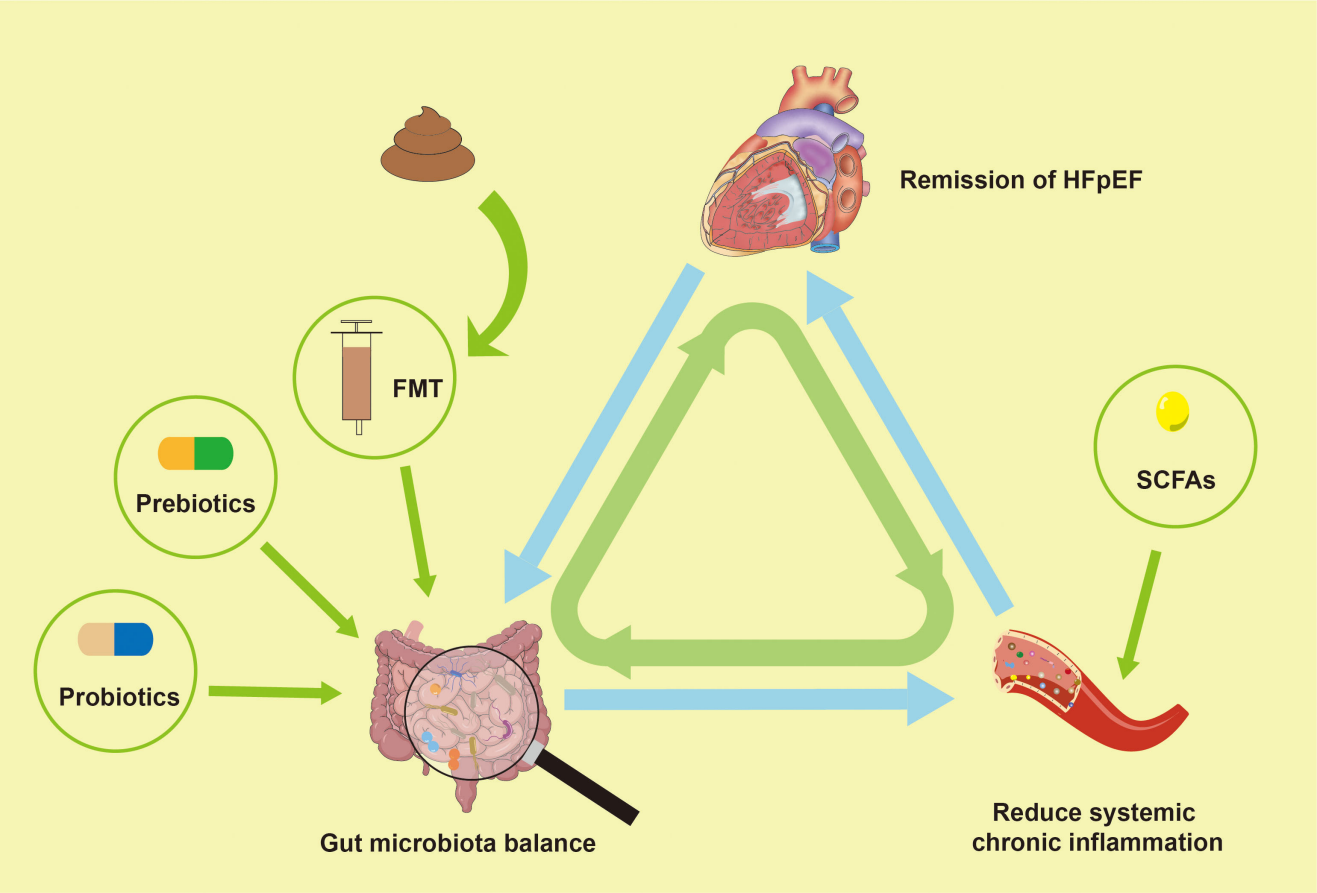
Modulating gut microbiota dysregulation may improve HFpEF. HFpEF, Heart failure with preserved left ventricular ejection fraction; FMT, fecal microbial transplantation; SCFAs, short-chain fatty acids.

In patients with HFpEF, gut microbiota dysregulation is prevalent and may positively impact pathophysiology by modulating gut microbiota or their metabolites through interventions such as probiotics, fecal microbial transplantation (FMT), prebiotics and replenishing gut microbiota metabolites (**Figure 4**). This has been confirmed in animal experiments (**Table 3**).

**Table 3:** Regulation of gut microbiota affects pathophysiological literature related to HFpEF.

Treatment	Model	Changes in gut microbiota	Cardiac changes
Dietary nitrate, FMT (Petrick et al., 2023)	C57BL/6N male mice	Decrease the abundance of *Firmicutes* and the ratio of *Firmicutes* to *Bacteroidetes*	Prevent high-fat diet–induced diastolic dysfunction
GN (Wang et al., 2022a)	male wild-type C57BL/6J mice	Decrease the abundance of *Firmicutes* and the ratio of *Firmicutes* to *Bacteroidetes*	Improve myocardial fibrosis/ventricular remodeling, reverse cardiomyocyte hypertrophy
Emodin (Evans et al., 2023)	C57BL/6 female mice	Increase the abundance of *Lachnospiraceae x4554*, *Ruminococcaeae*, *Akkermansia* and *Roseburia*.	Attenuated cardiac hypertrophy.
Astragaloside IV (Du et al., 2022)	C57BL/6J male mice	Increase the abundance of *Akkermansia*, *Defluviitaleaceae_UCG-011*, and *Rikenella*. Decrease the abundance of *Bacteroidetes*.	Ameliorated heart tissues inflammatory cell infiltration, myocardial fiber thickening, myocardial necrosis, and myocardial structural disorder.
Probiotics/prebiotics/synbiotics (Hu et al., 2022)	Wistar rats	Alter the abundances of *Ruminococcaceae* and *Lachnospiraceae*.	Attenuated cardiac hypertrophy.
Dapagliflozin (Bao et al., 2023)	C57BL/6N male mice	Decreased the ratio of *Firmicutes*/*Bacteroidetes*.	Reduced inflammation and cardiac fibrosis.
FMT (Zhang et al., 2022)	SD male rats	Decrease the ratio of *Firmicutes* to *Bacteroidetes*, and increase the abundance of *Enterobacteriaceae.*	Increase cardiac fibrosis.
Myricetin (Zhu et al., 2024)	C57BL/6J male mice	Increase the abundance of short-chain fatty acid-producing bacteria involving *Roseburia*, *Faecalibaculum*, and *Bifidobacterium*.	Decreasing cardiomyocyte hypertrophy and interstitial fibrosis.
Doxorubicin/FMT (An et al., 2021)	C57BL/6J male mice	Doxorubicin decreases abundance of *Prevotellaceae_UCG−001*, increases the abundance of *Alloprevotella* and *Rikenellaceae_RC9_gut_group*. FMT increased abundance of *Alloprevotella*, *Prevotellaceae_UCG−001* and *Rikenellaceae_RC9_gut_group*.	Doxorubicin induces cardiac pathological remodeling. FMT alleviates doxorubicin-induced cardiac fibrosis.
KTBA (Xu et al., 2024)	SD rats	Enrich the abundance of *Deltaproteobacteria*, *Desulfovibrionaceae*, and *Desulfovibrionales*.	Delays cardiomyocyte hypertrophy and fibrosis.
Aged FMT (Xu et al., 2024)	C57BL/6J mice	Decrease the abundance of *Firmicutes*, increase the abundance of *Bacteroidetes* and *Proteobacteria*.	promoting myocardial apoptosis, fibrosis and myocardial hypertrophy.
YHJF (Liu et al., 2022)	C57BL/6J mice	Reversed the increase in *Bacteroidetes* and the decrease in *Firmicutes* and *Ruminococcaceae*.	Reversal of left ventricle hypertrophy and myocardial fibrosis.
High-salt diet (Li et al., 2022)	C57BL/6J mice	Increase the abundance of *Burkholderiales bacterium YL45*, *Lactobacillus johnsonii*, and *Lactobacillus reuteri*.	Increased the HW/BW ratio, and cardiac hypertrophy/fibrosis.

FMT, fecal microbial transplantation; GN, ginseng dingzhi decoction; KTBA, kidney-tonifying blood-activating decoction; YHJF, yiqi-huoxue-jiangzhuo formula; HW, heart weight; BW, body weight.

### 6.1 Probiotics

The significant contribution of probiotics in maintaining human health and regulating immune function is significant (Wang et al., 2022b; Cristofori et al., 2021; Gavzy et al., 2023; Mazziotta et al., 2023; Bai et al., 2024). Clinical trials have demonstrated that individuals with cardiovascular disease can achieve significant improvements in cardiovascular health following approximately 12 weeks of probiotic therapy. Supplementation with *Lactobacillus paracaseis* is effective in controlling low-density lipoprotein cholesterol levels and delaying atherosclerosis progression in hyperlipidemic patients (Khongrum et al., 2023). In trials of probiotic interventions for coronary heart disease, inflammatory indicators such as LPS, IL-1β, hs-CRP, and TNF-α were significantly decreased in the probiotic group compared to the control group after 12 weeks of probiotic intervention (Moludi et al., 2021; Moludi et al., 2022).

Several studies have indicated that probiotics can enhance plasma markers indicative of inflammation and oxidative stress in HF, and significantly ameliorate HF-related comorbidities (Karim et al., 2022; Pourrajab et al., 2022; Cui et al., 2023). However, it is important to acknowledge that trials didn’t differentiate based on ejection fraction categories. Probiotics can also capable of modulating the metabolite production of gut microbiota (Sadeghi et al., 2023), which could have a positive impact on HFpEF and its complications. For instance, in obese mice on a high-fat diet, the administration of *Clostridium tyrobutyricum* notably improved gut microbiota dysregulation, increased the production of SCFAs, and reduced the intestinal expression of TNF-α, IL-6, IL-1β (Luo et al., 2024). Given that obesity is an important comorbidity in HFpEF, and a key factor in the pathogenesis of HFpEF, inflammation plays a central role in its development (Chirinos et al., 2020). These findings suggest that probiotics may exert beneficial effects on HFpEF by modulating the gut microbiota dysregulation. Further experimental research is needed to confirm these potential benefits.

### 6.2 Fecal microbial transplantation

FMT has garnered widespread attention as a therapeutic strategy for restoring gut microbiota ecological balance. Analogous to organ transplantation, FMT is the process of transplanting the fecal microbiota from a healthy donor into the gut of the patients with microbiota-related diseases (Leshem et al., 2019). The procedure aims to treat conditions by re-establishing a balanced microbial community. FMT has demonstrated the ability to improve atherosclerosis in genetically susceptible mice lacking C1q/TNF-associated protein (Kim et al., 2022), and it has shown a positive effect on cardiometabolic diseases (Pakmehr A, Mousavi et al., 2024). The therapeutic effect of FMT treatment may be associated with increased diversity of the gut microbiota in the recipient (Luqman et al., 2024).

A previous study reported that FMT in obese mice increased circulating butyrate levels (Bonomo et al., 2020). Recent studies have discovered that FMT from lean mice to obese pre-HFpEF mice augments early cardiac diastolic dysfunction and left ventricular hypertrophy, mechanisms consistent with the effects of butyrate therapy (Hatahet et al., 2023). Petrick et al. demonstrated that FMT of microorganisms from nitrate-fed mice into mice with cardiac dysfunction led to significant improvements in myocardial remodeling, oxidative stress, glucose intolerance, serum dyslipidemia and cardiac dysfunction. Additionally, this study indicated that cardiac protective effects of nitrates are associated with the remission of intestinal dysbiosis by FMT (Petrick et al., 2023). These experiments highlight the potential effect of gut microbiota modification to improve outcomes in HFpEF. The current effects demonstrated by FMT in animal trials may provide a novel approach for future clinical research.

### 6.3 Others

Prebiotics are non-digestible food ingredient that stimulate microbial growth, maintain a balanced gut ecosystem, thereby contributing to human health through the modulation of gut microbiota composition and function. Non-digestible plant oligosaccharides are the most popular prebiotics (Farias D de et al., 2019). Many studies have investigated the effects of prebiotics intake, including cellulose, on cardiovascular disease. Evidence suggests that prebiotics can inhibit the inflammatory response in healthy individuals by inducing the production of immunoregulatory molecules and lactic acid by the Bifidobacterium and Lactobacillus genera (Davani-Davari et al., 2019). Furthermore, dietary involving polysaccharide, flavonoid, and polyphenols can modulate gut microbiota, potentially affecting cardiovascular health (Feng et al., 2022). These processes may be related to metabolites such as BAs, SCFAs, AAs, etc (Xu and Yang, 2021).

Exogenous supplementation of gut microbiota metabolites, particularly SCFAs, may be an effective therapeutic strategy for HFpEF. Studies shown that SCFAs supplementation can increase acetic acid levels in the colon, and elevate the relative abundance of beneficial microbes such as *Akkermansia*, *Turicibacter*, *Ruminococcus*, and *Prevotella*, while also enhancing gut barrier function. SCFAs supplementation has been demonstrated to reduce the levels of NLRP3 and IL-1β, promote the IRF4/STAT3 complex and attenuate systemic inflammatory responses (Su et al., 2022), which may improve HFpEF. However, there is currently a lack of relevant clinical researches, and in light of the safety of approach, the large cohorts of clinical research should be conducted.

## 7 Discussion

Advances in research have enhanced our comprehension of HFpEF pathogenesis, especially the hypothesis that gut microbiota dysregulation significantly contributes to HFpEF etiology, which is gaining traction. This article focuses on the gut microbiota-inflammation-HFpEF axis in HFpEF, which has been shown to have a positive impact in relevant studies, and provides new strategies for future treatment of HFpEF. Despite the progress achieved with this strategy, current research on the gut microbiota-inflammation-HFpEF axis remains limited. We should expand large clinical cohort studies and further clinical and basic studies to elucidate potential mechanisms.

## References

[B1] An L. Wuri J. Zheng Z. Li W. Yan T. (2021). Microbiota modulate Doxorubicin induced cardiotoxicity. Eur. J. Pharm. Sci. 166, 105977. doi: 10.1016/j.ejps.2021.105977 34416387

[B2] Anselmi G. Gagliardi L. Egidi G. Leone S. Gasbarrini A. Miggiano G. A. D.. (2021). Gut microbiota and cardiovascular diseases: A critical review. Cardiol. Rev. 29, 195–204. doi: 10.1097/CRD.0000000000000327 32639240

[B3] Antar S. A. Ashour N. A. Marawan M. E. Al-Karmalawy A. A. (2023). Fibrosis: types, effects, markers, mechanisms for disease progression, and its relation with oxidative stress, immunity, and inflammation. Int. J. Mol. Sci. 24, 4004. doi: 10.3390/ijms24044004 36835428 PMC9963026

[B4] Bai J. Zhao X. Zhang M. Xia X. Yang A. Chen H. (2024). Gut microbiota: A target for prebiotics and probiotics in the intervention and therapy of food allergy. Crit. Rev. Food Sci. Nutr. 64, 3623–3637. doi: 10.1080/10408398.2022.2133079 36218372

[B5] Bal N. B. Han S. Kiremitci S. Sadi G. Uludag O. Demirel-Yilmaz E. (2019). Hypertension-induced cardiac impairment is reversed by the inhibition of endoplasmic reticulum stress. J. Pharm. Pharmacol. 71, 1809–1821. doi: 10.1111/jphp.13169 31579948

[B6] Bao N. Liu X. Zhong X. Jia S. Hua N. Zhang L.. (2023). Dapagliflozin-affected endothelial dysfunction and altered gut microbiota in mice with heart failure. Peerj. 11, e15589. doi: 10.7717/peerj.15589 37520255 PMC10386824

[B7] Bauernfeind F. G. Horvath G. Stutz A. Alnemri E. S. MacDonald K. Speert D.. (2009). Cutting edge: NF-kappaB activating pattern recognition and cytokine receptors license NLRP3 inflammasome activation by regulating NLRP3 expression. J. Immunol. (Baltimore Md: 1950). 183, 787–791. doi: 10.4049/jimmunol.0901363 PMC2824855 19570822

[B8] Beale A. L. O’Donnell J. A. Nakai M. E. Nanayakkara S. Vizi D. Carter K.. (2021). The gut microbiome of heart failure with preserved ejection fraction. J. Am. Heart Association: Cardiovasc. Cerebrovascular Disease. 10, e020654. doi: 10.1161/JAHA.120.020654 PMC8403331 34212778

[B9] Bent R. Moll L. Grabbe S. (2018). Interleukin-1 beta—a friend or foe in Malignancies? Int. J. Mol. Sci. 19, 2155. doi: 10.3390/ijms19082155 30042333 PMC6121377

[B10] Bezhaeva T. Karper J. Quax P. H. A. de Vries M. R. (2022). The intriguing role of TLR accessory molecules in cardiovascular health and disease. Front. Cardiovasc. Med. 9, 820962. doi: 10.3389/fcvm.2022.820962 35237675 PMC8884272

[B11] Bonomo R. R. Cook T. M. Gavini C. K. White C. R. Jones J. R. Bovo E.. (2020). Fecal transplantation and butyrate improve neuropathic pain, modify immune cell profile, and gene expression in the PNS of obese mice. Proc. Natl. Acad. Sci. U.S.A. 117, 26482–26493. doi: 10.1073/pnas.2006065117 33020290 PMC7584890

[B12] Bradley J. (2008). TNF-mediated inflammatory disease. J. Pathol. 214, 149–160. doi: 10.1002/path.v214:2 18161752

[B13] Caldarelli M. Rio P. Marrone A. Giambra V. Gasbarrini A. Gambassi G.. (2024). Inflammaging: the next challenge-exploring the role of gut microbiota, environmental factors, and sex differences. Biomedicines. 12, 1716. doi: 10.3390/biomedicines12081716 39200181 PMC11351301

[B14] Cao S. Zhang Q. Wang C. Wu H. Jiao L. Hong Q.. (2018). LPS challenge increased intestinal permeability, disrupted mitochondrial function and triggered mitophagy of piglets. Innate Immun. 24, 221–230. doi: 10.1177/1753425918769372 29642727 PMC6830921

[B15] Carrillo-Salinas F. J. Anastasiou M. Ngwenyama N. Kaur K. Tai A. Smolgovsky S. A.. (2020). Gut dysbiosis induced by cardiac pressure overload enhances adverse cardiac remodeling in a T cell-dependent manner. Gut Microbes 12, 1823801. doi: 10.1080/19490976.2020.1823801 33103561 PMC7588211

[B16] Chakaroun R. M. Olsson L. M. Bäckhed F. (2023). The potential of tailoring the gut microbiome to prevent and treat cardiometabolic disease. Nat. Rev. Cardiol. 20, 217–235. doi: 10.1038/s41569-022-00771-0 36241728

[B17] Challa A. A. Lewandowski E. D. (2022). Short-chain carbon sources: exploiting pleiotropic effects for heart failure therapy. JACC: Basic Trans. Sci. 7, 730–742. doi: 10.1016/j.jacbts.2021.12.010 PMC9357564 35958686

[B18] Chen S. Law C. S. Grigsby C. L. Olsen K. Hong T. T. Zhang Y.. (2011). Cardiomyocyte-specific deletion of the vitamin D receptor gene results in cardiac hypertrophy. Circulation. 124, 1838–1847. doi: 10.1161/CIRCULATIONAHA.111.032680 21947295 PMC4160312

[B19] Chen P. Pei J. Wang X. Tai S. Tang L. Hu X. (2022). Gut bacterial metabolite Urolithin A inhibits myocardial fibrosis through activation of Nrf2 pathway *in vitro* and *in vivo* . Mol. Med. 28, 19. doi: 10.1186/s10020-022-00444-1 35135471 PMC8822684

[B20] Chen K. Zheng X. Feng M. Li D. Zhang H. (2017). Gut microbiota-dependent metabolite trimethylamine N-oxide contributes to cardiac dysfunction in western diet-induced obese mice. Front. Physiol. 8, 139. doi: 10.3389/fphys.2017.00139 28377725 PMC5359299

[B21] Cheng K. C. Chang W. T. Kuo F. Y. Chen Z. C. Li Y. Cheng J. T. (2019). TGR5 activation ameliorates hyperglycemia-induced cardiac hypertrophy in H9c2 cells. Sci. Rep. 9, 3633. doi: 10.1038/s41598-019-40002-0 30842472 PMC6403401

[B22] Cheng X. Zhao H. Wen X. Li G. Guo S. Zhang D. (2023). NLRP3-inflammasome inhibition by MCC950 attenuates cardiac and pulmonary artery remodelling in heart failure with preserved ejection fraction. Life Sci. 333, 122185. doi: 10.1016/j.lfs.2023.122185 37858713

[B23] Chiang C. J. Tsai B. C. K. Lu T. L. Chao Y. P. Day C. H. Ho T. J.. (2021). Diabetes-induced cardiomyopathy is ameliorated by heat-killed Lactobacillus reuteri GMNL-263 in diabetic rats via the repression of the toll-like receptor 4 pathway. Eur. J. Nutr. 60, 3211–3223. doi: 10.1007/s00394-020-02474-z 33555373

[B24] Chirinos J. A. Akers S. R. Trieu L. Ischiropoulos H. Doulias P. Tariq A.. (2016). Heart failure, left ventricular remodeling, and circulating nitric oxide metabolites. JAHA. 5, e004133. doi: 10.1161/JAHA.116.004133 27742619 PMC5121510

[B25] Chirinos J. A. Orlenko A. Zhao L. Basso M. D. Cvijic M. E. Li Z.. (2020). Multiple plasma biomarkers for risk stratification in patients with heart failure and preserved ejection fraction. J. Am. Coll. Cardiol. 75, 1281–1295. doi: 10.1016/j.jacc.2019.12.069 32192654 PMC7147356

[B26] Cristofori F. Dargenio V. N. Dargenio C. Miniello V. L. Barone M. Francavilla R. (2021). Anti-inflammatory and immunomodulatory effects of probiotics in gut inflammation: A door to the body. Front. Immunol. 12, 578386. doi: 10.3389/fimmu.2021.578386 33717063 PMC7953067

[B27] Cui H. Han S. Dai Y. Xie W. Zheng R. Sun Y. (2023). Gut microbiota and integrative traditional Chinese and western medicine in prevention and treatment of heart failure. Phytomedicine 117, 154885. doi: 10.1016/j.phymed.2023.154885 37302262

[B28] Dahlem C. Kado S. Y. He Y. Bein K. Wu D. Haarmann-Stemmann T.. (2020). AHR signaling interacting with nutritional factors regulating the expression of markers in vascular inflammation and atherogenesis. Int. J. Mol. Sci. 21, 8287. doi: 10.3390/ijms21218287 33167400 PMC7663825

[B29] Davani-Davari D. Negahdaripour M. Karimzadeh I. Seifan M. Mohkam M. Masoumi S. J.. (2019). Prebiotics: definition, types, sources, mechanisms, and clinical applications. Foods. 8, 92. doi: 10.3390/foods8030092 30857316 PMC6463098

[B30] Dawuti A. Sun S. Wang R. Gong D. (2023). Salvianolic acid A alleviates heart failure with preserved ejection fraction via regulating TLR/Myd88/TRAF/NF-κB and p38MAPK/CREB signaling pathways. BioMed. Pharmacother. 168, 115837. doi: 10.1016/j.biopha.2023.115837 37931518

[B31] Deng Y. Xie M. Li Q. Xu X. Ou W. Zhang Y.. (2021). Targeting mitochondria-inflammation circuit by β-hydroxybutyrate mitigates HFpEF. Circ. Res. 128, 232–245. doi: 10.1161/CIRCRESAHA.120.317933 33176578

[B32] Dinarello C. A. (2011). Interleukin-1 in the pathogenesis and treatment of inflammatory diseases. Blood. 117, 3720–3732. doi: 10.1182/blood-2010-07-273417 21304099 PMC3083294

[B33] Dong T. Huang D. Jin Z. (2024). Mechanism of sodium butyrate, a metabolite of gut microbiota, regulating cardiac fibroblast transdifferentiation via the NLRP3/Caspase-1 pyroptosis pathway. J. Cardiothorac Surg. 19, 208. doi: 10.1186/s13019-024-02692-0 38616256 PMC11017590

[B34] Dong Z. Zheng S. Shen Z. Luo Y. Hai X. (2021). Trimethylamine N-oxide is associated with heart failure risk in patients with preserved ejection fraction. Lab. Med. 52, 346–351. doi: 10.1093/labmed/lmaa075 33135738

[B35] Drapkina O. M. Ashniev G. A. Zlobovskaya O. A. Yafarova A. A. Dementeva E. V. Kaburova A. N.. (2022). Diversities in the gut microbial patterns in patients with atherosclerotic cardiovascular diseases and certain heart failure phenotypes. Biomedicines. 10, 2762. doi: 10.3390/biomedicines10112762 36359282 PMC9687836

[B36] Du X. Q. Shi L. P. Chen Z. W. Hu J. Y. Zuo B. Xiong Y.. (2022). Astragaloside IV ameliorates isoprenaline-induced cardiac fibrosis in mice via modulating gut microbiota and fecal metabolites. Front. Cell Infect. Microbiol. 12, 836150. doi: 10.3389/fcimb.2022.836150 35656031 PMC9152365

[B37] Eblimit Z. Thevananther S. Karpen S. J. Taegtmeyer H. Moore D. D. Adorini L.. (2018). TGR5 activation induces cytoprotective changes in the heart and improves myocardial adaptability to physiologic, inotropic, and pressure-induced stress in mice. Cardiovasc. Ther. 36, e12462. doi: 10.1111/cdr.2018.36.issue-5 30070769 PMC6800140

[B38] Evans L. Price T. Hubert N. Moore J. Shen Y. Athukorala M.. (2023). Emodin inhibited pathological cardiac hypertrophy in response to angiotensin-induced hypertension and altered the gut microbiome. Biomolecules. 13, 1274. doi: 10.3390/biom13091274 37759673 PMC10526847

[B39] Farias D de P. de Araújo F. F. Neri-Numa I. A. Pastore G. M. (2019). Prebiotics: Trends in food, health and technological applications. Trends Food Sci. Technol. 93, 23–35. doi: 10.1016/j.tifs.2019.09.004

[B40] Fayyaz A. U. Eltony M. Prokop L. J. Koepp K. E. Borlaug B. A. Dasari S.. (2024). Pathophysiological insights into HFpEF from studies of human cardiac tissue. Nat. Rev. Cardiol. 28, 1–15. doi: 10.1038/s41569-024-01067-1 PMC11750620 39198624

[B41] Feng W. Liu J. Cheng H. Zhang D. Tan Y. Peng C. (2022). Dietary compounds in modulation of gut microbiota-derived metabolites. Front. Nutr. 9, 939571. doi: 10.3389/fnut.2022.939571 35928846 PMC9343712

[B42] Franssen C. Chen S. Unger A. Korkmaz H. I. De Keulenaer G. W. Tschöpe C.. (2016). Myocardial microvascular inflammatory endothelial activation in heart failure with preserved ejection fraction. JACC Heart failure. 4, 312–324. doi: 10.1016/j.jchf.2015.10.007 26682792

[B43] Gavzy S. J. Kensiski A. Lee Z. L. Mongodin E. F. Ma B. Bromberg J. S. (2023). Bifidobacterium mechanisms of immune modulation and tolerance. Gut Microbes 15, 2291164. doi: 10.1080/19490976.2023.2291164 38055306 PMC10730214

[B44] Gojda J. Cahova M. (2021). Gut microbiota as the link between elevated BCAA serum levels and insulin resistance. Biomolecules. 11, 1414. doi: 10.3390/biom11101414 34680047 PMC8533624

[B45] Grebe A. Hoss F. Latz E. (2018). NLRP3 inflammasome and the IL-1 pathway in atherosclerosis. Circ. Res. 122, 1722–1740. doi: 10.1161/CIRCRESAHA.118.311362 29880500

[B46] Grishanova A. Y. Perepechaeva M. L. (2024). Kynurenic acid/ahR signaling at the junction of inflammation and cardiovascular diseases. Int. J. Mol. Sci. 25, 6933. doi: 10.3390/ijms25136933 39000041 PMC11240928

[B47] Guan X. Q. Wang C. H. Cheng P. Fu L. Y. Wu Q. J. Cheng G.. (2023). Effects of empagliflozin on gut microbiota in heart failure with a preserved ejection fraction: the design of a pragmatic randomized, open-label controlled trial (EMPAGUM). Drug Design Dev. Ther. 17, 1495–1502. doi: 10.2147/DDDT.S404479 PMC10202117 37223722

[B48] Guo F. Qiu X. Tan Z. Li Z. Ouyang D. (2020). Plasma trimethylamine n-oxide is associated with renal function in patients with heart failure with preserved ejection fraction. BMC Cardiovasc. Disord. 20, 394. doi: 10.1186/s12872-020-01669-w 32859154 PMC7456383

[B49] Güven B. Sun Q. Wagg C. S. Almeida de Oliveira A. Silver H. Persad K. L.. (2024). Obesity is a major determinant of impaired cardiac energy metabolism in heart failure with preserved ejection fraction. J. Pharmacol. Exp. Ther. 388, 145–155. doi: 10.1124/jpet.123.001791 37977817

[B50] Hamo C. E. DeJong C. Hartshorne-Evans N. Lund L. H. Shah S. J. Solomon S.. (2024). Heart failure with preserved ejection fraction. Nat. Rev. Dis. Primers. 10, 1–19. doi: 10.1038/s41572-024-00540-y 39143132

[B51] Han K. Meadows A. M. Rodman M. J. Russo A. C. Sharma R. Singh K.. (2024). Propionate functions as a feeding state–dependent regulatory metabolite to counter proinflammatory signaling linked to nutrient load and obesity. J. Leukocyte Biol. 115, 738-749. doi: 10.1093/jleuko/qiae006 38207130 PMC10980352

[B52] Han J. Ye S. Zou C. Chen T. Wang J. Li J.. (2018). Angiotensin II causes biphasic STAT3 activation through TLR4 to initiate cardiac remodeling. Hypertension (Dallas Tex: 1979). 72, 1301–1311. doi: 10.1161/HYPERTENSIONAHA.118.11860 30571233

[B53] Hanna A. Frangogiannis N. G. (2020). Inflammatory cytokines and chemokines as therapeutic targets in heart failure. Cardiovasc. Drugs Ther. 34, 849–863. doi: 10.1007/s10557-020-07071-0 32902739 PMC7479403

[B54] Hatahet J. Cook T. M. Bonomo R. R. Elshareif N. Gavini C. K. White C. R.. (2023). Fecal microbiome transplantation and tributyrin improves early cardiac dysfunction and modifies the BCAA metabolic pathway in a diet induced pre-HFpEF mouse model. Front. Cardiovasc. Med. 10, 1105581. doi: 10.3389/fcvm.2023.1105581 36844730 PMC9944585

[B55] Heinzel F. R. Hohendanner F. Jin G. Sedej S. Edelmann F. (2015). Myocardial hypertrophy and its role in heart failure with preserved ejection fraction. J. Appl. Physiol. (1985) 119, 1233–1242. doi: 10.1152/japplphysiol.00374.2015 26183480 PMC4652334

[B56] Hietbrink F. Besselink M. G. H. Renooij W. de Smet M. B. M. Draisma A. van der Hoeven H.. (2009). Systemic inflammation increases intestinal permeability during experimental human endotoxemia. Shock (Augusta Ga). 32, 374–378. doi: 10.1097/SHK.0b013e3181a2bcd6 19295480

[B57] Hou K. Wu Z. X. Chen X. Y. Wang J. Q. Zhang D. Xiao C.. (2022). Microbiota in health and diseases. Signal Transduction Targeted Ther. 7, 135. doi: 10.1038/s41392-022-00974-4 PMC9034083 35461318

[B58] Hu Y. Pan Z. Huang Z. Li Y. Han N. Zhuang X.. (2022). Gut microbiome-targeted modulations regulate metabolic profiles and alleviate altitude-related cardiac hypertrophy in rats. Microbiol. Spectr. 10, e01053–e01021. doi: 10.1128/spectrum.01053-21 PMC8826942 35138162

[B59] Huang Z. Mei X. Jiang Y. Chen T. Zhou Y. (2021). Gut microbiota in heart failure patients with preserved ejection fraction (GUMPTION study). Front. Cardiovasc. Med. 8, 803744. doi: 10.3389/fcvm.2021.803744 35071367 PMC8770938

[B60] Hubesch G. Hanthazi A. Acheampong A. Chomette L. Lasolle H. Hupkens E.. (2022). A preclinical rat model of heart failure with preserved ejection fraction with multiple comorbidities. Front. Cardiovasc. Med. 8, 809885. doi: 10.3389/fcvm.2021.809885 35097026 PMC8793630

[B61] Hummel S. L. Bassis C. Marolt C. Konerman M. Schmidt T. M. (2019). Gut microbiome differs between heart failure with preserved ejection fraction and age-matched controls. J. Am. Coll. Cardiol. 73, 750. doi: 10.1016/S0735-1097(19)31358-0

[B62] Janeiro M. H. Ramírez M. J. Milagro F. I. Martínez J. A. Solas M. (2018). Implication of trimethylamine N-oxide (TMAO) in disease: Potential biomarker or new therapeutic target. Nutrients 10, 13998. doi: 10.33909/nu10101398 PMC6213249 30275434

[B63] Jeong J. Choe J. (2023). Akt, IL-4, and STAT proteins play distinct roles in prostaglandin production in human follicular dendritic cell-like cells. Int. J. Mol. Sci. 24, 16692. doi: 10.3390/ijms242316692 38069015 PMC10706142

[B64] Joseph P. Dokainish H. McCready T. Budaj A. Roy A. Ertl G.. (2020). A multinational registry to study the characteristics and outcomes of heart failure patients: The global congestive heart failure (G-CHF) registry. Am. Heart J. 227, 56–63. doi: 10.1016/j.ahj.2020.06.002 32679282

[B65] Kaburova A. N. Drapkina O. M. Uydin S. M. Pokrovskaya M. S. Koretsky S. N. Efimova I. A.. (2020a). The relationship between the key markers of myocardial fibrosis and gut microbiota composition in patients with heart failure and preserved ejection fraction. Eur. Heart J. 41, ehaa946.0861. doi: 10.1093/ehjci/ehaa946.0861

[B66] Kaburova A. N. Drapkina O. M. Uydin S. M. Vishnyakova M. V. Pokrovskaya M. S. Koretsky S. N.. (2020b). The link between left ventricular extracellular volume determined by T1 myocardial mapping and gut microbiota composition in individuals with heart failure and preserved ejection fraction. Eur. Heart J. 41, ehaa946.0864. doi: 10.1093/ehjci/ehaa946.0864

[B67] Kaburova A. N. Drapkina O. M. Yudin S. M. Koretsky S. N. Makarov V. V. Pokrovskaya M. S.. (2021). Relationship between gut microbiota and markers of myocardial fibrosis in with chronic heart failure with preserved ejection fraction. Cardiovasc. Ther. Prev. 20, 2834. doi: 10.15829/1728-8800-2021-2834

[B68] Kaburova A. N. Poyarkov S. V. Makarov V. V. Drapkina O. M. Uydin S. M. Pokrovskaya M. S.. (2019). Serum Trimethylamine N-Oxide levels and 16S rRNA gut microbiota profiling in patients with heart failure and preserved ejection fraction and healthy individuals. Eur. Heart J. 40, ehz747.0060. doi: 10.1093/eurheartj/ehz747.0060

[B69] Kakkar R. Lee R. T. (2008). The IL-33/ST2 pathway: therapeutic target and novel biomarker. Nat. Rev. Drug Discovery. 7, 827–840. doi: 10.1038/nrd2660 18827826 PMC4277436

[B70] Kapur N. K. (2011). Transforming growth factor-β: governing the transition from inflammation to fibrosis in heart failure with preserved left ventricular function. Circ. Heart Failure. 4, 5–7. doi: 10.1161/CIRCHEARTFAILURE.110.960054 21245455

[B71] Karim A. Muhammad T. Shah I. Khan J. Qaisar R. (2022). A multistrain probiotic reduces sarcopenia by modulating Wnt signaling biomarkers in patients with chronic heart failure. J. Cardiol. 80, 449–455. doi: 10.1016/j.jjcc.2022.06.006 35750555

[B72] Kaye D. M. Shihata W. A. Jama H. A. Tsyganov K. Ziemann M. Kiriazis H.. (2020). Deficiency of prebiotic fiber and insufficient signaling through gut metabolite-sensing receptors leads to cardiovascular disease. Circulation. 141, 1393–1403. doi: 10.1161/CIRCULATIONAHA.119.043081 32093510

[B73] Khongrum J. Yingthongchai P. Boonyapranai K. Wongtanasarasin W. Aobchecy P. Tateing S.. (2023). Safety and effects of lactobacillus paracasei TISTR 2593 Supplementation on improving cholesterol metabolism and atherosclerosis-related parameters in subjects with hypercholesterolemia: A randomized, double-blind, placebo-controlled clinical trial. Nutrients. 15, 661. doi: 10.3390/nu15030661 36771367 PMC9921995

[B74] Kim E. S. Yoon B. H. Lee S. M. Choi M. Kim E. H. Lee B. W.. (2022). Fecal microbiota transplantation ameliorates atherosclerosis in mice with C1q/TNF-related protein 9 genetic deficiency. Exp. Mol. Med. 54, 103–114. doi: 10.1038/s12276-022-00728-w 35115674 PMC8894390

[B75] Kinugasa Y. Nakamura K. Kamitani H. Hirai M. Yanagihara K. Kato M.. (2021). Trimethylamine N-oxide and outcomes in patients hospitalized with acute heart failure and preserved ejection fraction. ESC Heart Failure. 8, 2103. doi: 10.1002/ehf2.13290 33734604 PMC8120352

[B76] Koufou E. E. Assimakopoulos S. F. Bosgana P. de Lastic A. L. Grypari I. M. Georgopoulou G. A.. (2024). Altered expression of intestinal tight junction proteins in heart failure patients with reduced or preserved ejection fraction: A pathogenetic mechanism of intestinal hyperpermeability. Biomedicines. 12, 160. doi: 10.3390/biomedicines12010160 38255265 PMC10813326

[B77] Krittanawong C. Britt W. M. Rizwan A. Siddiqui R. Khawaja M. Khan R.. (2024). Clinical update in heart failure with preserved ejection fraction. Curr. Heart Fail Rep. 21, 461–484. doi: 10.1007/s11897-024-00679-5 39225910

[B78] Lebedev D. A. Lyasnikova E. A. Vasilyeva E. Y. Babenko A. Y. Shlyakhto E. V. (2020). Type 2 diabetes mellitus and chronic heart failure with midrange and preserved ejection fraction: A focus on serum biomarkers of fibrosis. J. Diabetes Res. 2020, 6976153. doi: 10.1155/2020/6976153 33224989 PMC7669344

[B79] Lekawanvijit S. Adrahtas A. Kelly D. J. Kompa A. R. Wang B. H. Krum H. (2010). Does indoxyl sulfate, a uraemic toxin, have direct effects on cardiac fibroblasts and myocytes? Eur. Heart J. 31, 1771–1779. doi: 10.1093/eurheartj/ehp574 20047993

[B80] Leshem A. Horesh N. Elinav E. (2019). Fecal microbial transplantation and its potential application in cardiometabolic syndrome. Front. Immunol. 10, 1341. doi: 10.3389/fimmu.2019.01341 31258528 PMC6587678

[B81] Li S. Chen S. Nie M. Wen L. Zou B. Zhang L.. (2022). Salt-sensitive ileal microbiota plays a role in atrial natriuretic peptide deficiency-induced cardiac injury. Nutrients. 14, 3129. doi: 10.3390/nu14153129 35956306 PMC9370783

[B82] Li J. Lü H. Chen S. (2022). Trimethylamine oxide induces pyroptosis of vascular endothelial cells through ALDH2/ROS/NLRP3/GSDMD pathway. J. Cent. South Univ. Med. Sci. 47, 1171–1181. doi: 10.11817/j.issn.1672-7347.2022.220086 PMC10930322 36411700

[B83] Li C. Qin D. Hu J. Yang Y. Hu D. Yu B. (2022). Inflamed adipose tissue: A culprit underlying obesity and heart failure with preserved ejection fraction. Front. Immunol. 13, 947147. doi: 10.3389/fimmu.2022.947147 36483560 PMC9723346

[B84] Li T. Wang Y. Liu C. Hu Y. Wu M. Li J.. (2009). MyD88-dependent nuclear factor-kappaB activation is involved in fibrinogen-induced hypertrophic response of cardiomyocytes. J. Hypertens. 27, 1084–1093. doi: 10.1097/HJH.0b013e3283293c93 19342961

[B85] Li Z. Wu Z. Yan J. Liu H. Liu Q. Deng Y.. (2019). Gut microbe-derived metabolite trimethylamine N-oxide induces cardiac hypertrophy and fibrosis. Lab. Invest. 99, 346–357. doi: 10.1038/s41374-018-0091-y 30068915

[B86] Libby P. (2021). Targeting inflammatory pathways in cardiovascular disease: The inflammasome, interleukin-1, interleukin-6 and beyond. Cells 10, 951. doi: 10.3390/cells10040951 33924019 PMC8073599

[B87] Lin C. J. Cheng Y. C. Chen H. C. Chao Y. K. Nicholson M. W. Yen E. C. L.. (2022). Commensal gut microbiota-derived acetate and propionate enhance heart adaptation in response to cardiac pressure overload in mice. Theranostics. 12, 7319–7334. doi: 10.7150/thno.76002 36438501 PMC9691357

[B88] Liu Z. G. Hsu H. Goeddel D. V. Karin M. (1996). Dissection of TNF receptor 1 effector functions: JNK activation is not linked to apoptosis while NF-kappaB activation prevents cell death. Cell. 87, 565–576. doi: 10.1016/S0092-8674(00)81375-6 8898208

[B89] Liu Y. Hu Z. F. Liao H. H. Liu W. Liu J. Ma Z. G.. (2015). Toll-like receptor 5 deficiency attenuates interstitial cardiac fibrosis and dysfunction induced by pressure overload by inhibiting inflammation and the endothelial–mesenchymal transition. Biochim. Biophys. Acta (BBA) - Mol. Basis Disease. 1852, 2456–2466. doi: 10.1016/j.bbadis.2015.08.013 26300483

[B90] Liu T. Lu X. Gao W. Zhai Y. Li H. Li S.. (2022). Cardioprotection effect of Yiqi–Huoxue–Jiangzhuo formula in a chronic kidney disease mouse model associated with gut microbiota modulation and NLRP3 inflammasome inhibition. BioMed. Pharmacother. 152, 113159. doi: 10.1016/j.biopha.2022.113159 35661533

[B91] Liu H. Magaye R. Kaye D. M. Wang B. H. (2024). Heart failure with preserved ejection fraction: The role of inflammation. Eur. J. Pharmacol. 980, 176858. doi: 10.1016/j.ejphar.2024.176858 39074526

[B92] Liu X. Shao Y. Tu J. Sun J. Li L. Tao J.. (2021). Trimethylamine-N-oxide-stimulated hepatocyte-derived exosomes promote inflammation and endothelial dysfunction through nuclear factor-kappa B signaling. Ann. Trans. Med. 9, 1670–1670. doi: 10.21037/atm-21-5043 PMC8667148 34988179

[B93] Lotierzo M. Dupuy A. M. Kalmanovich E. Roubille F. (2020). sST2 as a value-added biomarker in heart failure. Clin. Chim. Acta 501, 120–130. doi: 10.1016/j.cca.2019.10.029 31678574

[B94] Lund A. Nordrehaug J. E. Slettom G. Hafstad Solvang S. E. Ringdal Pedersen E. K. Midttun Ø.. (2020). Plasma kynurenines and prognosis in patients with heart failure. PLoS One 15, e0227365. doi: 10.1371/journal.pone.0227365 31923223 PMC6953806

[B95] Luo Y. Jin Y. Wang H. Wang G. Lin Y. Chen H.. (2024). Effects of clostridium tyrobutyricum on lipid metabolism, intestinal barrier function, and gut microbiota in obese mice induced by high-fat diet. Nutrients. 16, 493. doi: 10.3390/nu16040493 38398817 PMC10893108

[B96] Luqman A. Hassan A. Ullah M. Naseem S. Ullah M. Zhang L.. (2024). Role of the intestinal microbiome and its therapeutic intervention in cardiovascular disorder. Front. Immunol. 15, 1321395. doi: 10.3389/fimmu.2024.1321395 38343539 PMC10853344

[B97] Ma J. Piao X. Mahfuz S. Long S. Wang J. (2022). The interaction among gut microbes, the intestinal barrier and short chain fatty acids. Anim. Nutr. (Zhongguo Xu Mu Shou Yi Xue Hui). 9, 159–174. doi: 10.1016/j.aninu.2021.09.012 PMC9079705 35573092

[B98] Mahenthiran A. Wilcox J. Tang W. H. W. (2024). Heart failure: a punch from the gut. Curr. Heart Fail Rep. 21, 73–80. doi: 10.1007/s11897-024-00648-y 38300390 PMC10924029

[B99] Makrecka-Kuka M. Volska K. Antone U. Vilskersts R. Grinberga S. Bandere D.. (2017). Trimethylamine N-oxide impairs pyruvate and fatty acid oxidation in cardiac mitochondria. Toxicol. Lett. 267, 32–38. doi: 10.1016/j.toxlet.2016.12.017 28049038

[B100] Malesza I. J. Malesza M. Walkowiak J. Mussin N. Walkowiak D. Aringazina R.. (2021). High-fat, western-style diet, systemic inflammation, and gut microbiota: A narrative review. Cells. 10, 3164. doi: 10.3390/cells10113164 34831387 PMC8619527

[B101] Mamic P. Chaikijurajai T. Tang W. H. W. (2021). Gut microbiome - A potential mediator of pathogenesis in heart failure and its comorbidities: state-of-the-art review. J. Mol. Cell. Cardiol. 152, 105–117. doi: 10.1016/j.yjmcc.2020.12.001 33307092 PMC7981261

[B102] Mamic P. Snyder M. Tang W. H. W. (2023). Gut microbiome-based management of patients with heart failure: JACC review topic of the week. J. Am. Coll. Cardiol. 81, 1729–1739. doi: 10.1016/j.jacc.2023.02.045 37100490

[B103] Martin F. P. J. Wang Y. Sprenger N. Yap I. K. S. Lundstedt T. Lek P.. (2008). Probiotic modulation of symbiotic gut microbial–host metabolic interactions in a humanized microbiome mouse model. Mol. Syst. Biol. 4, 157. doi: 10.1038/msb4100190 18197175 PMC2238715

[B104] Martins D. Silva C. Ferreira A. C. Dourado S. Albuquerque A. Saraiva F.. (2024). Unravelling the gut microbiome role in cardiovascular disease: A systematic review and a meta-analysis. Biomolecules. 14, 731. doi: 10.3390/biom14060731 38927134 PMC11201797

[B105] Masenga S. K. Povia J. P. Lwiindi P. C. Kirabo A. (2023). Recent advances in microbiota-associated metabolites in heart failure. Biomedicines. 11, 2313. doi: 10.3390/biomedicines11082313 37626809 PMC10452327

[B106] Mazziotta C. Tognon M. Martini F. Torreggiani E. Rotondo J. C. (2023). Probiotics mechanism of action on immune cells and beneficial effects on human health. Cells. 12, 184. doi: 10.3390/cells12010184 36611977 PMC9818925

[B107] McGarrah R. W. White P. J. (2023). Branched-chain amino acids in cardiovascular disease. Nat. Rev. Cardiol. 20, 77–89. doi: 10.1038/s41569-022-00760-3 36064969 PMC10284296

[B108] Mishra S. Kass D. A. (2021). Cellular and molecular pathobiology of heart failure with preserved ejection fraction. Nat. Rev. Cardiol. 18, 400–423. doi: 10.1038/s41569-020-00480-6 33432192 PMC8574228

[B109] Modrego J. Ortega-Hernández A. Goirigolzarri J. Restrepo-Córdoba M. A. Bäuerl C. Cortés-Macías E.. (2023). Gut microbiota and derived short-chain fatty acids are linked to evolution of heart failure patients. Int. J. Mol. Sci. 24, 13892. doi: 10.3390/ijms241813892 37762194 PMC10530267

[B110] Mohr A. E. Crawford M. Jasbi P. Fessler S. Sweazea K. L. (2022). Lipopolysaccharide and the gut microbiota: considering structural variation. FEBS letters. 596, 849–875. doi: 10.1002/1873-3468.14328 35262962

[B111] Moludi J. Kafil H. S. Qaisar S. A. Gholizadeh P. Alizadeh M. Vayghyan H. J. (2021). Effect of probiotic supplementation along with calorie restriction on metabolic endotoxemia, and inflammation markers in coronary artery disease patients: a double blind placebo controlled randomized clinical trial. Nutr. J. 20, 47. doi: 10.1186/s12937-021-00703-7 34074289 PMC8170788

[B112] Moludi J. Khedmatgozar H. Nachvak S. M. Abdollahzad H. Moradinazar M. Sadeghpour tabaei A. (2022). The effects of co-administration of probiotics and prebiotics on chronic inflammation, and depression symptoms in patients with coronary artery diseases: a randomized clinical trial. Nutr. Neurosci. 25, 1659–1668. doi: 10.1080/1028415X.2021.1889451 33641656

[B113] Mongirdienė A. Liobikas J. (2022). Phenotypic and functional heterogeneity of monocyte subsets in chronic heart failure patients. Biology. 11, 195. doi: 10.3390/biology11020195 35205062 PMC8869357

[B114] Murphy T. M. Waterhouse D. F. James S. Casey C. Fitzgerald E. O’Connell E.. (2017). A comparison of HFrEF vs HFpEF’s clinical workload and cost in the first year following hospitalization and enrollment in a disease management program. Int. J. Cardiol. 232, 330–335. doi: 10.1016/j.ijcard.2016.12.057 28087180

[B115] Museedi A. S. Samson R. Le Jemtel T. H. (2024). Menopause, epicardial adiposity and preserved ejection fraction heart failure. Int. J. Cardiol. 415, 132478. doi: 10.1016/j.ijcard.2024.132478 39179034

[B116] Nair N. (2020). Epidemiology and pathogenesis of heart failure with preserved ejection fraction. Rev. Cardiovasc. Med. 21, 531–540. doi: 10.31083/j.rcm.2020.04.154 33387998

[B117] Nemet I. Li X. S. Haghikia A. Li L. Wilcox J. Romano K. A.. (2023). Atlas of gut microbe-derived products from aromatic amino acids and risk of cardiovascular morbidity and mortality. Eur. Heart J. 44, 3085–3096. doi: 10.1093/eurheartj/ehad333 37342006 PMC10481777

[B118] O’Donovan A. N. Herisson F. M. Fouhy F. Ryan P. M. Whelan D. Johnson C. N.. (2020). Gut microbiome of a porcine model of metabolic syndrome and HF-pEF. Am. J. Physiol. Heart Circulatory Physiol. 318, H590–H603. doi: 10.1152/ajpheart.00512.2019 32031871

[B119] Obokata M. Reddy Y. N. V. Borlaug B. A. (2020). Diastolic dysfunction and heart failure with preserved ejection fraction: understanding mechanisms with non-invasive methods. JACC Cardiovasc. Imaging 13, 245–257. doi: 10.1016/j.jcmg.2018.12.034 31202759 PMC6899218

[B120] Olsen M. B. Gregersen I. Sandanger Ø. Yang K. Sokolova M. Halvorsen B. E.. (2021). Targeting the inflammasome in cardiovascular disease. JACC: Basic to Trans. Science. 7, 84–98. doi: 10.1016/j.jacbts.2021.08.006 PMC8807732 35128212

[B121] Omote K. Verbrugge F. H. Borlaug B. A. (2022). Heart failure with preserved ejection fraction: mechanisms and treatment strategies. Annu. Rev. Med. 73, 321–337. doi: 10.1146/annurev-med-042220-022745 34379445 PMC9002335

[B122] Organ C. L. Li Z. Sharp T. E. Polhemus D. J. Gupta N. Goodchild T. T.. (2020). Nonlethal inhibition of gut microbial trimethylamine N-oxide production improves cardiac function and remodeling in a murine model of heart failure. J. Am. Heart Association: Cardiovasc. Cerebrovascular Disease. 9, e016223. doi: 10.1161/JAHA.119.016223 PMC7660847 32390485

[B123] Pakmehr A. Mousavi S. M. Ejtahed H. S. Hoseini-Tavassol Z. Siadat S. D. Hasani-Ranjbar S.. (2024). The effect of fecal microbiota transplantation on cardiometabolic risk factors: A systematic review and meta-analysis. Clin. Ther. 46, e87–100. doi: 10.1016/j.clinthera.2023.11.015 38087724

[B124] Park B. S. Lee J. O. (2013). Recognition of lipopolysaccharide pattern by TLR4 complexes. Exp. Mol. Med. 45, e66. doi: 10.1038/emm.2013.97 24310172 PMC3880462

[B125] Park J. J. Yoon M. Cho H. W. Cho H. J. Kim K. H. Yang D. H.. (2022). C-reactive protein and statins in heart failure with reduced and preserved ejection fraction. Front. Cardiovasc. Med. 9, 1064967. doi: 10.3389/fcvm.2022.1064967 36620625 PMC9816146

[B126] Paulus W. J. Tschöpe C. (2013). A novel paradigm for heart failure with preserved ejection fraction: comorbidities drive myocardial dysfunction and remodeling through coronary microvascular endothelial inflammation. J. Am. Coll. Cardiol. 62, 263–271. doi: 10.1016/j.jacc.2013.02.092 23684677

[B127] Perticone M. Gigliotti S. Shehaj E. Maio R. Suraci E. Miceli S.. (2024). Gut permeability and immune-mediated inflammation in heart failure. Biomedicines. 12, 1217. doi: 10.3390/biomedicines12061217 38927424 PMC11200601

[B128] Petrick H. L. Ogilvie L. M. Brunetta H. S. Robinson A. Kirsh A. J. Barbeau P. A.. (2023). Dietary nitrate and corresponding gut microbiota prevent cardiac dysfunction in obese mice. Diabetes. 72, 844–856. doi: 10.2337/db22-0575 36812497

[B129] Petruzziello C. Saviano A. Manetti L. L. Macerola N. Ojetti V. (2024). The role of gut microbiota and the potential effects of probiotics in heart failure. Medicina (Kaunas Lithuania). 60, 271. doi: 10.3390/medicina60020271 38399558 PMC10890346

[B130] Pourrajab B. Naderi N. Janani L. Hajahmadi M. Mofid V. Dehnad A.. (2022). The impact of probiotic yogurt versus ordinary yogurt on serum sTWEAK, sCD163, ADMA, LCAT and BUN in patients with chronic heart failure: a randomized, triple-blind, controlled trial. J. Sci. Food Agric. 102, 6024–6035. doi: 10.1002/jsfa.v102.13 35460085

[B131] Randeni N. Bordiga M. Xu B. (2024). A comprehensive review of the triangular relationship among diet-gut microbiota-inflammation. Int. J. Mol. Sci. 25, 9366. doi: 10.3390/ijms25179366 39273314 PMC11394685

[B132] Rani S. Sreenivasaiah P. K. Kim J. O. Lee M. Y. Kang W. S. Kim Y. S.. (2017). Tauroursodeoxycholic acid (TUDCA) attenuates pressure overload-induced cardiac remodeling by reducing endoplasmic reticulum stress. PLoS One 12, e0176071. doi: 10.1371/journal.pone.0176071 28426781 PMC5398705

[B133] Razquin C. Ruiz-Canela M. Toledo E. Hernández-Alonso P. Clish C. B. Guasch-Ferré M.. (2021). Metabolomics of the tryptophan–kynurenine degradation pathway and risk of atrial fibrillation and heart failure: potential modification effect of mediterranean diet. Am. J. Clin. Nutr. 114, 1646–1654. doi: 10.1093/ajcn/nqab238 34291275 PMC8764340

[B134] Rodrigues-e-Lacerda R. Fang H. Robin N. Bhatwa A. Marko D. M. Schertzer J. D. (2023). Microbiota and Nod-like receptors balance inflammation and metabolism during obesity and diabetes. Biomed. J. 46, 100610. doi: 10.1016/j.bj.2023.100610 37263539 PMC10505681

[B135] Romano K. A. Vivas E. I. Amador-Noguez D. Rey F. E. (2015). Intestinal microbiota composition modulates choline bioavailability from diet and accumulation of the proatherogenic metabolite trimethylamine-N-oxide. mBio. 6, e02481–e02414. doi: 10.1128/mBio.02481-14 25784704 PMC4453578

[B136] Rose-John S. (2020). Interleukin-6 signalling in health and disease. F1000research. 9, 1013. doi: 10.12688/f1000research PMC7443778 32864098

[B137] Ryu J. K. Kim S. J. Rah S. H. Kang J. I. Jung H. E. Lee D.. (2017). Reconstruction of LPS transfer cascade reveals structural determinants within LBP, CD14, and TLR4-MD2 for efficient LPS recognition and transfer. Immunity. 46, 38–50. doi: 10.1016/j.immuni.2016.11.007 27986454

[B138] Sadeghi A. Ebrahimi M. Kharazmi M. S. Jafari S. M. (2023). Effects of microbial-derived biotics (meta/pharma/post-biotics) on the modulation of gut microbiome and metabolome; general aspects and emerging trends. Food Chem. 411, 135478. doi: 10.1016/j.foodchem.2023.135478 36696721

[B139] Salzano A. Israr M. Z. Yazaki Y. Heaney L. M. Kanagala P. Singh A.. (2020). Combined use of trimethylamine N-oxide with BNP for risk stratification in heart failure with preserved ejection fraction: findings from the DIAMONDHFpEF study. Eur. J. Prev. Cardiol. 27, 2159–2162. doi: 10.1177/2047487319870355 31412713

[B140] Sanders-van-Wijk S. Tromp J. Beussink-Nelson L. Hage C. Svedlund S. Saraste A.. (2020). Proteomic evaluation of the comorbidity-inflammation paradigm in heart failure with preserved ejection fraction: results from the PROMIS-HFpEF study. Circulation. 142, 2029–2044. doi: 10.1161/CIRCULATIONAHA.120.045810 33034202 PMC7686082

[B141] Sanders-van-Wijk S. van Empel V. Davarzani N. Maeder M. T. Handschin R. Pfisterer M. E.. (2015). Circulating biomarkers of distinct pathophysiological pathways in heart failure with preserved vs. reduced left ventricular ejection fraction. Eur. J. Heart Fail. 17, 1006–1014. doi: 10.1002/ejhf.2015.17.issue-10 26472682

[B142] Savarese G. Becher P. M. Lund L. H. Seferovic P. Rosano G. M. C. Coats A. J. S. (2022). Global burden of heart failure: a comprehensive and updated review of epidemiology. Cardiovasc. Res. 118, 3272–3287. doi: 10.1093/cvr/cvac013 35150240

[B143] Schiattarella G. G. Rodolico D. Hill J. A. (2021). Metabolic inflammation in heart failure with preserved ejection fraction. Cardiovasc. Res. 117, 423–434. doi: 10.1093/cvr/cvaa217 32666082 PMC8599724

[B144] Shah S. J. Kitzman D. W. Borlaug B. A. van Heerebeek L. Zile M. R. Kass D. A.. (2016). Phenotype-specific treatment of heart failure with preserved ejection fraction: A multiorgan roadmap. Circulation. 134, 73–90. doi: 10.1161/CIRCULATIONAHA.116.021884 27358439 PMC4930115

[B145] Sharma S. Garg I. Ashraf M. Z. (2016). TLR signalling and association of TLR polymorphism with cardiovascular diseases. Vasc. Pharmacol. 87, 30–37. doi: 10.1016/j.vph.2016.10.008 27826031

[B146] Shi M. Wei J. Yuan H. Li Y. Guo Z. (2023). The role of the gut microbiota and bile acids in heart failure: A review. Med. (baltimore). 102, e35795. doi: 10.1097/MD.0000000000035795 PMC10637566 37960774

[B147] Shi B. Zhang X. Song Z. Dai Z. Luo K. Chen B.. (2023).Targeting gut microbiota–derived kynurenine to predict and protect the remodeling of the pressure-overloaded young heart. Sci. Adv. 9, eadg7417. doi: 10.1126/sciadv.adg7417 37450589 PMC10348671

[B148] Shimazu S. Hirashiki A. Okumura T. Yamada T. Okamoto R. Shinoda N.. (2013). Association between indoxyl sulfate and cardiac dysfunction and prognosis in patients with dilated cardiomyopathy. Circ. Journal: Off. J. Japanese Circ. Society. 77, 390–396. doi: 10.1253/circj.CJ-12-0715 23100090

[B149] Singh M. V. Swaminathan P. D. Luczak E. D. Kutschke W. Weiss R. M. Anderson M. E. (2012). MyD88 mediated inflammatory signaling leads to CaMKII oxidation, cardiac hypertrophy and death after myocardial infarction. J. Mol. Cell Cardiol. 52, 1135–1144. doi: 10.1016/j.yjmcc.2012.01.021 22326848 PMC3327770

[B150] Stolfi C. Maresca C. Monteleone G. Laudisi F. (2022). Implication of intestinal barrier dysfunction in gut dysbiosis and diseases. Biomedicines. 10, 289. doi: 10.3390/biomedicines10020289 35203499 PMC8869546

[B151] Su S. H. Wu Y. F. Lin Q. Zhang L. Wang D. P. Hai J. (2022). Fecal microbiota transplantation and replenishment of short-chain fatty acids protect against chronic cerebral hypoperfusion-induced colonic dysfunction by regulating gut microbiota, differentiation of Th17 cells, and mitochondrial energy metabolism. J. Neuroinflamm. 19, 313. doi: 10.1186/s12974-022-02675-9 PMC9791754 36567333

[B152] Sun X. Jiao X. Ma Y. Liu Y. Zhang L. He Y.. (2016). Trimethylamine N-oxide induces inflammation and endothelial dysfunction in human umbilical vein endothelial cells via activating ROS-TXNIP-NLRP3 inflammasome. Biochem. Biophys. Res. Commun. 481, 63–70. doi: 10.1016/j.bbrc.2016.11.017 27833015

[B153] Sun G. Yin Z. Liu N. Bian X. Yu R. Su X.. (2017). Gut microbial metabolite TMAO contributes to renal dysfunction in a mouse model of diet-induced obesity. Biochem. Biophys. Res. Commun. 493, 964–970. doi: 10.1016/j.bbrc.2017.09.108 28942145

[B154] Suzuki T. Yazaki Y. Voors A. A. Jones D. J. L. Chan D. C. S. Anker S. D.. (2019). Association with outcomes and response to treatment of trimethylamine N-oxide in heart failure: results from BIOSTAT-CHF. Eur. J. Heart Fail. 21, 877–886. doi: 10.1002/ejhf.2019.21.issue-7 30370976

[B155] Tang W. H. W. Backhed F. Landmesser U. Hazen S. L. (2019). Intestinal microbiota in cardiovascular health and disease: JACC state-of-the-art review. J. Am. Coll. Cardiol. 73, 2089–2105. doi: 10.1016/j.jacc.2019.03.024 31023434 PMC6518422

[B156] Tang T. W. H. Chen H. C. Chen C. Y. Yen C. Y. T. Lin C. J. Prajnamitra R. P.. (2019). Loss of gut microbiota alters immune system composition and cripples postinfarction cardiac repair. Circulation. 139, 647–659. doi: 10.1161/CIRCULATIONAHA.118.035235 30586712

[B157] Tang M. Yin Y. Wang W. Gong K. Dong J. (2024). Exploring the multifaceted effects of Interleukin-1 in lung cancer: From tumor development to immune modulation. Life Sci. 342, 122539. doi: 10.1016/j.lfs.2024.122539 38423172

[B158] Taslim N. A. Yusuf M. Ambari A. M. Del Rosario Puling I. M. Ibrahim F. Z. Hardinsyah H.. (2023). Anti-inflammatory, antioxidant, metabolic and gut microbiota modulation activities of Probiotic in cardiac remodeling condition: evidence from systematic study and meta-analysis of randomized controlled trials. Probiotics Antimicrobial Proteins. 15, 1049–1061. doi: 10.1007/s12602-023-10105-2 37349622 PMC10393865

[B159] Telesca M. De Angelis A. Donniacuo M. Bellocchio G. Riemma M. A. Mele E.. (2024). Effects of sacubitril-valsartan on aging-related cardiac dysfunction. Eur. J. Pharmacol. 978, 176794. doi: 10.1016/j.ejphar.2024.176794 38968980

[B160] Ter Maaten J. M. Damman K. Verhaar M. C. Paulus W. J. Duncker D. J. Cheng C.. (2016). Connecting heart failure with preserved ejection fraction and renal dysfunction: the role of endothelial dysfunction and inflammation. Eur. J. Heart Fail. 18, 588–598. doi: 10.1002/ejhf.2016.18.issue-6 26861140

[B161] Thomas T. P. Grisanti L. A. (2020). The dynamic interplay between cardiac inflammation and fibrosis. Front. Physiol. 11, 529075. doi: 10.3389/fphys.2020.529075 33041853 PMC7522448

[B162] Tishkoff D. X. Nibbelink K. A. Holmberg K. H. Dandu L. Simpson R. U. (2008). Functional vitamin D receptor (VDR) in the T-tubules of cardiac myocytes: VDR knockout cardiomyocyte contractility. Endocrinology. 149, 558–564. doi: 10.1210/en.2007-0805 17974622 PMC2219302

[B163] Trøseid M. Ueland T. Hov J. R. Svardal A. Gregersen I. Dahl C. P.. (2015). Microbiota-dependent metabolite trimethylamine-N-oxide is associated with disease severity and survival of patients with chronic heart failure. J. Intern. Med. 277, 717–726. doi: 10.1111/joim.12328 25382824

[B164] Turdi S. Hu N. Ren J. (2013). Tauroursodeoxycholic acid mitigates high fat diet-induced cardiomyocyte contractile and intracellular ca2+ Anomalies. PLoS One 8, e63615. doi: 10.1371/journal.pone.0063615 23667647 PMC3647067

[B165] Vasavan T. Ferraro E. Ibrahim E. Dixon P. Gorelik J. Williamson C. (2018). Heart and bile acids – Clinical consequences of altered bile acid metabolism. Biochim. Biophys. Acta (BBA) - Mol. Basis Dis. 1864, 1345–1355. doi: 10.1016/j.bbadis.2017.12.039 29317337

[B166] Violi F. Cammisotto V. Bartimoccia S. Pignatelli P. Carnevale R. Nocella C. (2023). Gut-derived low-grade endotoxaemia, atherothrombosis and cardiovascular disease. Nat. Rev. Cardiol. 20, 24–37. doi: 10.1038/s41569-022-00737-2 35840742 PMC9284488

[B167] Wan Y. Yuan J. Li J. Li H. Zhang J. Tang J.. (2020). Unconjugated and secondary bile acid profiles in response to higher-fat, lower-carbohydrate diet and associated with related gut microbiota: A 6-month randomized controlled-feeding trial. Clin. Nutr. (Edinburgh Scotland). 39, 395–404. doi: 10.1016/j.clnu.2019.02.037 30876827

[B168] Wang J. Chen P. Cao Q. Wang W. Chang X. (2022a). Traditional chinese medicine ginseng dingzhi decoction ameliorates myocardial fibrosis and high glucose-induced cardiomyocyte injury by regulating intestinal flora and mitochondrial dysfunction. Oxid. Med. Cell Longev. 2022, 9205908. doi: 10.1155/2022/9205908 35401934 PMC8989614

[B169] Wang X. He G. Peng Y. Zhong W. Wang Y. Zhang B. (2015). Sodium butyrate alleviates adipocyte inflammation by inhibiting NLRP3 pathway. Sci. Rep. 5, 12676. doi: 10.1038/srep12676 26234821 PMC4522654

[B170] Wang Y. C. Koay Y. C. Pan C. Zhou Z. Tang W. Wilcox J.. (2024). Indole-3-propionic acid protects against heart failure with preserved ejection fraction. Circ. Res. 134, 371–389. doi: 10.1161/CIRCRESAHA.123.322381 38264909 PMC10923103

[B171] Wang G. Kong B. Shuai W. Fu H. Jiang X. Huang H. (2020). 3,3-Dimethyl-1-butanol attenuates cardiac remodeling in pressure-overload-induced heart failure mice. J. Nutr. Biochem. 78, 108341. doi: 10.1016/j.jnutbio.2020.108341 32004931

[B172] Wang Z. Liu C. Wei J. Yuan H. Shi M. Zhang F.. (2024). Network and experimental pharmacology on mechanism of yixintai regulates the TMAO/PKC/NF-κB signaling pathway in treating heart failure. Drug Design Dev. Ther. 18, 1415–1438. doi: 10.2147/DDDT.S448140 PMC11069381 38707614

[B173] Wang J. Qie J. Zhu D. Zhang X. Zhang Q. Xu Y.. (2022b). The landscape in the gut microbiome of long-lived families reveals new insights on longevity and aging – relevant neural and immune function. Gut Microbes 14, 2107288. doi: 10.1080/19490976.2022.2107288 35939616 PMC9361766

[B174] Wang H. B. Wang P. Y. Wang X. Wan Y. L. Liu Y. C. (2012). Butyrate enhances intestinal epithelial barrier function via up-regulation of tight junction protein Claudin-1 transcription. Digestive Dis. Sci. 57, 3126–3135. doi: 10.1007/s10620-012-2259-4 22684624

[B175] Wang W. Wu L. Du X. Zhang F. Ullah S. H. Lei T.. (2019). Anti-Toll-like receptor 2 antibody inhibits nuclear factor kappa B activation and attenuates cardiac damage in high-fat-feeding rats. Acta Biochim. Et Biophys. Sinica. 51, 347–355. doi: 10.1093/abbs/gmz009 30877771

[B176] Wang J. Zhu N. Su X. Gao Y. Yang R. (2023). Gut-microbiota-derived metabolites maintain gut and systemic immune homeostasis. Cells. 12, 793. doi: 10.3390/cells12050793 36899929 PMC10000530

[B177] Weber A. Wasiliew P. Kracht M. (2010). Interleukin-1 (IL-1) pathway. Sci. Signal 3, cm1. doi: 10.1126/scisignal.3105cm1 20086235

[B178] Wen S. He L. Zhong Z. Zhao R. Weng S. Mi H.. (2021). Stigmasterol restores the balance of treg/th17 cells by activating the butyrate-PPARγ Axis in colitis. Front. Immunol. 12, 741934. doi: 10.3389/fimmu.2021.741934 34691046 PMC8526899

[B179] Wen Y. Sun Z. Xie S. Hu Z. Lan Q. Sun Y.. (2022). Intestinal flora derived metabolites affect the occurrence and development of cardiovascular disease. J. Multidiscip. Healthcare. 15, 2591–2603. doi: 10.2147/JMDH.S367591 PMC9656419 36388628

[B180] Wenzl F. A. Ambrosini S. Mohammed S. A. Kraler S. Lüscher T. F. Costantino S.. (2021). Inflammation in metabolic cardiomyopathy. Front. Cardiovasc. Med. 8, 742178. doi: 10.3389/fcvm.2021.742178 34671656 PMC8520939

[B181] Westermann D. Lindner D. Kasner M. Zietsch C. Savvatis K. Escher F.. (2011). Cardiac inflammation contributes to changes in the extracellular matrix in patients with heart failure and normal ejection fraction. Circ. Heart Failure. 4, 44–52. doi: 10.1161/CIRCHEARTFAILURE.109.931451 21075869

[B182] Wilson Tang W. H. Wang Z. Fan Y. Levison B. Hazen J. E. Donahue L. M.. (2014). Prognostic value of elevated levels of intestinal microbe-generated metabolite trimethylamine-N-oxide in patients with heart failure: Refining the gut hypothesis. J. Am. Coll. Cardiol. 64, 1908–1914. doi: 10.1016/j.jacc.2014.02.617 25444145 PMC4254529

[B183] Wilson Tang W. H. Wang Z. Shrestha K. Borowski A. G. Wu Y. Troughton R. W.. (2015). Intestinal microbiota-dependent phosphatidylcholine metabolites, diastolic dysfunction and adverse clinical outcomes in chronic systolic heart failure. J. cardiac failure. 21, 91–96. doi: 10.1016/j.cardfail.2014.11.006 25459686 PMC4312712

[B184] Wu C. K. Lee J. K. Chiang F. T. Yang C. H. Huang S. W. Hwang J. J.. (2011). Plasma levels of tumor necrosis factor-α and interleukin-6 are associated with diastolic heart failure through downregulation of sarcoplasmic reticulum Ca2+ ATPase. Crit. Care Med. 39, 984–992. doi: 10.1097/CCM.0b013e31820a91b9 21263314

[B185] Xiaoli L. Zhen Z. Jiuchang Z. Hongjiang W. Xinchun Y. (2020). Activation of FXR receptor reduces damage of ET-1 on H9C2 cardiomyocytes by activating AMPK signaling pathway. Panminerva Med. 66, 137–145. doi: 10.23736/S0031-0808.20.03930-0 32414227

[B186] Xu R. Bi Y. He X. Zhang Y. Zhao X. (2024). Kidney-tonifying blood-activating decoction delays ventricular remodeling in rats with chronic heart failure by regulating gut microbiota and metabolites and p38 mitogen-activated protein kinase/p65 nuclear factor kappa-B/aquaporin-4 signaling pathway. J. Ethnopharmacol. 330, 118110. doi: 10.1016/j.jep.2024.118110 38580189

[B187] Xu H. Li O. Kim D. Xue M. Bao Z. Yang F. (2024). Aged microbiota exacerbates cardiac failure by PPARα/PGC1α pathway. Biochim. Biophys. Acta (BBA) - Mol. Basis Disease. 1870, 167271. doi: 10.1016/j.bbadis.2024.167271 38823462

[B188] Xu J. Yang Y. (2021). Gut microbiome and its meta-omics perspectives: profound implications for cardiovascular diseases. Gut Microbes 13, 1936379. doi: 10.1080/19490976.2021.1936379 34170211 PMC8237965

[B189] Yamamoto M. Seo Y. Ishizua T. Nakagawa D. Sato K. (2021). Comparison of soluble ST2, pentraxin-3, galectin-3, and high-sensitivity troponin T of cardiovascular outcomes in patients with acute decompensated heart failure. J. Card Fail 27, 1240–1250. doi: 10.1016/j.cardfail.2021.05.025 34129951

[B190] Yang Z. He M. Austin J. Sayed D. Abdellatif M. (2023). Reducing branched-chain amino acids improves cardiac stress response in mice by decreasing histone H3K23 propionylation. J. Clin. Invest. 133, e169399. doi: 1172.169399/JCI37669116 37669116 10.1172/JCI169399 PMC10645387

[B191] Yang H. J. Kong B. Shuai W. Zhang J. J. Huang H. (2020). Knockout of MD1 contributes to sympathetic hyperactivity and exacerbates ventricular arrhythmias following heart failure with preserved ejection fraction via NLRP3 inflammasome activation. Exp. Physiol. 105, 966–978. doi: 10.1113/eph.v105.6 32240565

[B192] Yang C. Li X. Hu M. Li T. Jiang L. Zhang Y. (2024). Gut microbiota as predictive biomarker for chronic heart failure in patients with different nutritional risk. J. Cardiovasc. Trans. Res. 17, 1240–1257. doi: 10.1007/s12265-024-10529-3 38913293

[B193] Yang H. Zhu J. Fu H. Shuai W. (2024). Dapansutrile ameliorates atrial inflammation and vulnerability to atrial fibrillation in HFpEF rats. Heart Lung Circ. 33, 65–77. doi: 10.1016/j.hlc.2023.09.017 38040503

[B194] Ye S. Lin K. Wu G. Xu M. J. Shan P. Huang W.. (2021). Toll-like receptor 2 signaling deficiency in cardiac cells ameliorates Ang II-induced cardiac inflammation and remodeling. Trans. Research: J. Lab. Clin. Med. 233, 62–76. doi: 10.1016/j.trsl.2021.02.011 33652137

[B195] Yntema T. Koonen D. P. Y. Kuipers F. (2023). Emerging roles of gut microbial modulation of bile acid composition in the etiology of cardiovascular diseases. Nutrients. 15, 1850. doi: 10.3390/nu15081850 37111068 PMC10141989

[B196] Yokota A. Fukiya S. Islam K. B. M. S. Ooka T. Ogura Y. Hayashi T.. (2012). Is bile acid a determinant of the gut microbiota on a high-fat diet? Gut Microbes 3, 455–459. doi: 10.4161/gmic.21216 22825495

[B197] Yu L. Feng Z. (2018). The role of toll-like receptor signaling in the progression of heart failure. Mediators Inflamm. 2018, 9874109. doi: 10.1155/2018/9874109 29576748 PMC5822798

[B198] Yuan L. Li Y. Chen M. Xue L. Wang J. Ding Y.. (2024). Therapeutic applications of gut microbes in cardiometabolic diseases: current state and perspectives. Appl. Microbiol. Biotechnol. 108, 156. doi: 10.1007/s00253-024-13007-7 38244075 PMC10799778

[B199] Yukino-Iwashita M. Nagatomo Y. Kawai A. Taruoka A. Yumita Y. Kagami K.. (2022). Short-chain fatty acids in gut–heart axis: their role in the pathology of heart failure. J. Personalized Med. 12, 1805. doi: 10.3390/jpm12111805 PMC9695649 36579524

[B200] Zhang X. Li Y. Yang P. Liu X. Lu L. Chen Y.. (2020). Trimethylamine-N-oxide promotes vascular calcification through activation of NLRP3 (Nucleotide-binding domain, leucine-rich-containing family, pyrin domain-containing-3) inflammasome and NF-κB (Nuclear factor κB) signals. Arteriosclerosis Thrombosis Vasc. Biol. 40, 751–765. doi: 10.1161/ATVBAHA.119.313414 31941382

[B201] Zhang Y. Wu J. Dong E. Wang Z. Xiao H. (2023). Toll-like receptors in cardiac hypertrophy. Front. Cardiovasc. Med. 10, 1143583. doi: 10.3389/fcvm.2023.1143583 37113698 PMC10126280

[B202] Zhang Y. Zhang S. Li B. Luo Y. Gong Y. Jin X.. (2022). Gut microbiota dysbiosis promotes age-related atrial fibrillation by lipopolysaccharide and glucose-induced activation of NLRP3-inflammasome. Cardiovasc. Res. 118, 785–797. doi: 10.1093/cvr/cvab114 33757127

[B203] Zhang S. Zhou J. Wu W. Zhu Y. Liu X. (2023). The role of bile acids in cardiovascular diseases: from mechanisms to clinical implications. Aging Disease. 14, 261–282. doi: 10.14336/AD.2022.0817 37008052 PMC10017164

[B204] Zhao M. Zhang J. Xu Y. Liu J. Ye J. Wang Z.. (2021). Selective inhibition of NLRP3 inflammasome reverses pressure overload-induced pathological cardiac remodeling by attenuating hypertrophy, fibrosis, and inflammation. Int. Immunopharmacol. 99, 108046. doi: 10.1016/j.intimp.2021.108046 34435581

[B205] Zhou W. Anakk S. (2022). Enterohepatic and non-canonical roles of farnesoid X receptor in controlling lipid and glucose metabolism. Mol. Cell. endocrinology. 549, 111616. doi: 10.1016/j.mce.2022.111616 PMC9245558 35304191

[B206] Zhu J. Bao Z. Hu Z. Wu S. Tian C. Zhou Y.. (2024). Myricetin alleviates diabetic cardiomyopathy by regulating gut microbiota and their metabolites. Nutr. Diabetes. 14, 10. doi: 10.1038/s41387-024-00268-4 38472186 PMC10933338

